# Active Nuclear Import of Mammalian Cell-Expressible DNA Origami

**DOI:** 10.1021/jacs.2c12733

**Published:** 2023-02-24

**Authors:** Anna Liedl, Johanna Grießing, Jessica A. Kretzmann, Hendrik Dietz

**Affiliations:** † Department of Biosciences, School of Natural Sciences, Technical University of Munich, Am Coulombwall 4a, 85748 Garching, Germany; ‡ Munich Institute of Biomedical Engineering, Technical University of Munich, Boltzmannstraße 11, 85748 Garching, Germany

## Abstract

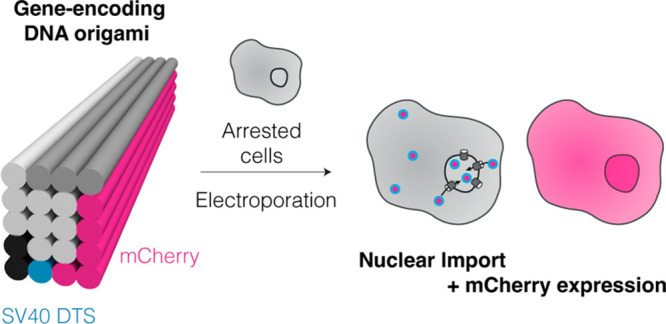

DNA origami enables the creation of complex 3D shapes from genetic material. Future uses could include the delivery of genetic instructions to cells, but nuclear import remains a major barrier to gene delivery due to the impermeability of the nuclear membrane. Here we realize active nuclear import of DNA origami objects in dividing and chemically arrested mammalian cells. We developed a custom DNA origami single-strand scaffold featuring a mammalian-cell expressible reporter gene (mCherry) and multiple Simian virus 40 (SV40) derived DNA nuclear targeting sequences (DTS). Inclusion of the DTS within DNA origami rescued gene expression in arrested cells, indicating that active transport into the nucleus occurs. Our work successfully adapts mechanisms known from viruses to promote the cellular expression of genetic instructions encoded within DNA origami objects.

The delivery of custom genetic instructions to cells continues to drive major fundamental and therapeutic advances. Genetic instructions are typically delivered as linear or circularized double-stranded (ds) DNA or RNA or as single-stranded (ss) DNA or RNA.^1−4^ Since programmable self-assembly with DNA or RNA origami enables the fabrication of complex three-dimensional objects from DNA or RNA in a user-defined manner,^5−8^ these approaches could also enable delivering genetic instructions in more complex ways and with additional functionalities to cells.

Nuclear import remains a major barrier to successful gene delivery due to the impermeability of the nuclear membrane.^9^ It is predominantly hypothesized that delivered genetic material passively enters the nucleus during mitosis when the nuclear envelope breaks down.^10,11^ However, this passive entry is relatively inefficient, and only a small amount of the delivered DNA reaches the nucleus.^12^ Furthermore, most cells *in vivo* are either postmitotic (nondividing) or have very long doubling times, limiting entry via passive nuclear transport during mitosis.^12,13^

Previous work explored the use of proteins engineered with nuclear localization signals (NLS) in complex with DNA origami objects to aid the nuclear import.^14^ Herein, we have designed and investigated DNA origami objects that directly encode instructions for enhancing active nuclear import—so-called DNA nuclear targeting sequences (DTS). In addition, the objects contain mammalian-cell expressible genetic instructions in the form of an mCherry reporter gene expression cassette to enable a fluorescent read-out for quantifying access to the nucleus (Figure 1a). To investigate delivery in both dividing and nondividing cells, we employed human embryonic kidney cells (HEK293T, default doubling time ∼20 h), with and without chemical cell cycle arrest.

**Figure 1:**
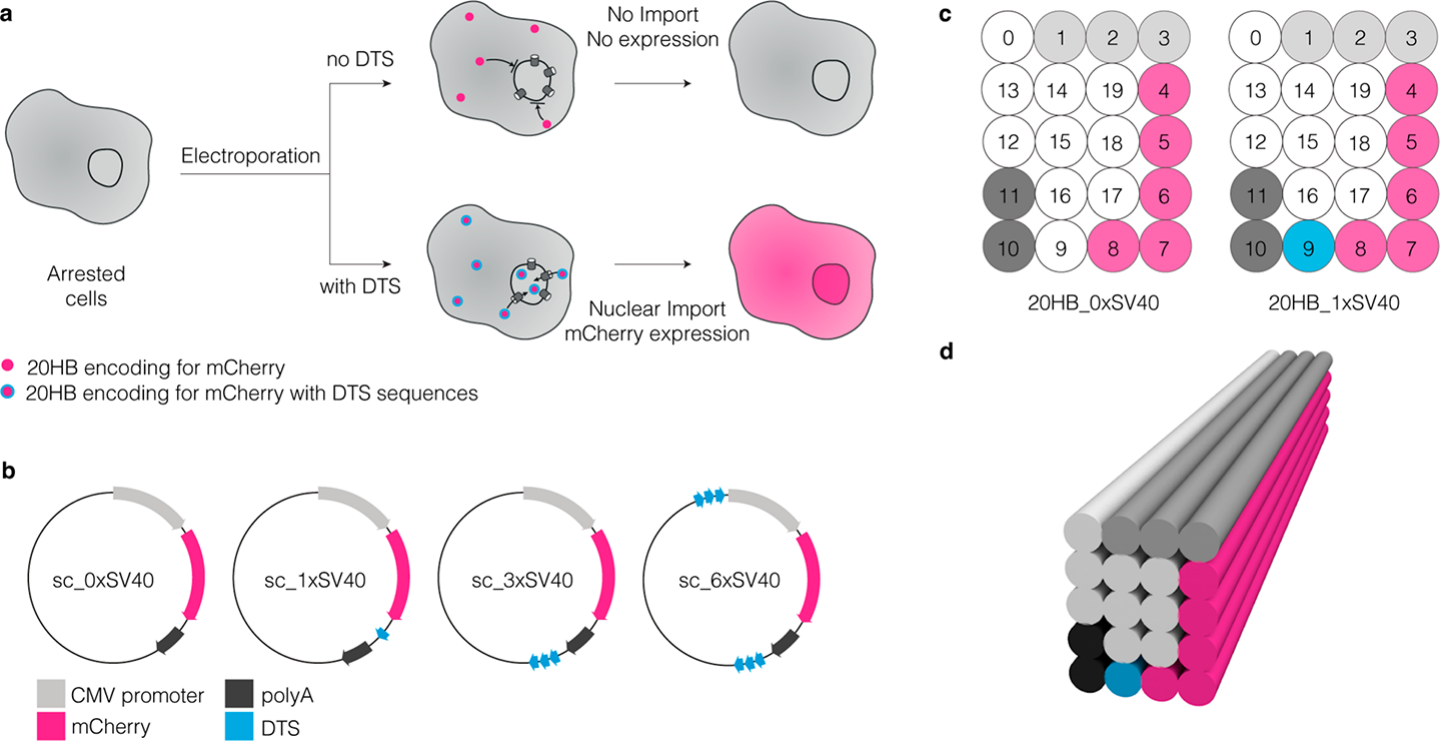
Engineering nuclear localization signals into custom DNA origami scaffolds and structures. (a) Electroporation enables delivery of DNA origami directly to the cytoplasm. Cellular recognition of DTS sequences should enhance nuclear uptake and thus expression of mCherry in nondividing cells, which are modeled here using a chemically arrested cell model. (b) Plasmid designs with varying numbers of SV40 DTS sequences included (0×, 1×, 3×, and 6×SV40 repeats) for production of custom ssDNA scaffolds. (c) Schematic cross section of helices (0–19) for the 20HB structures displaying sequences of interest in the exterior helices. (d) Cylindrical model of the used DNA origami structure, a 20-helix bundle (20HB). The colored regions in (b), (c), and (d) display the gene encoded on the scaffold part in the respective helix.

For construction of the custom scaffolds featuring DTS, we utilized previously described plasmid backbones compatible with bacteriophage production of ssDNA.^15^ The custom ssDNA scaffold was designed to include a CMV promoter, mCherry reporter gene, and a bGH polyA signal, to be encoded in the 5′ to 3′ direction (coding strand). We chose to use the 72 bp Simian virus 40 (SV40) DTS,^16^ and incorporated either zero, one, three, or six repeats of the SV40 DTS sequence (0×, 1×, 3×, or 6×SV40) into the scaffold sequence design (Figure 1b). Multiple distinct transcription factors are known to bind to the SV40 DTS sequence within the cytoplasm, and then direct transportation to the nucleus.^17,18^ To exploit this pathway, we designed a 20-helix bundle (20HB) test object in which 20 helices are packed on a square lattice, and routed the scaffold strand such that the gene features and DTS sequences are displayed on the exterior helices of the objects (Figure 1c,d, Figure S1). We chose a 20HB design as a simple design which is both compact and elongated, which has been previously demonstrated to be beneficial to cellular uptake.^19^

We produced the custom ssDNA scaffolds and characterized them by gel electrophoresis against previously verified ssDNA markers (Figure 2a and Figure S2). The scaffolds including 0×, 1×, 3×, and 6×SV40 repeats are sized 4363 nt, 4086 nt, 4453 nt, and 4741 nt, respectively, and sequences are included in Supporting Information. Four DNA origami objects were folded and purified, one from each of the custom scaffolds (Figure 2a,b). The 20HB_0×SV40 and 20HB_1×SV40 folded at good yield with a clear leading band and without major side products, while the 20HB_3×SV40 and 20HB_6×SV40 displayed impurities in the gel-electrophoretic analysis (Figure S3).

**Figure 2:**
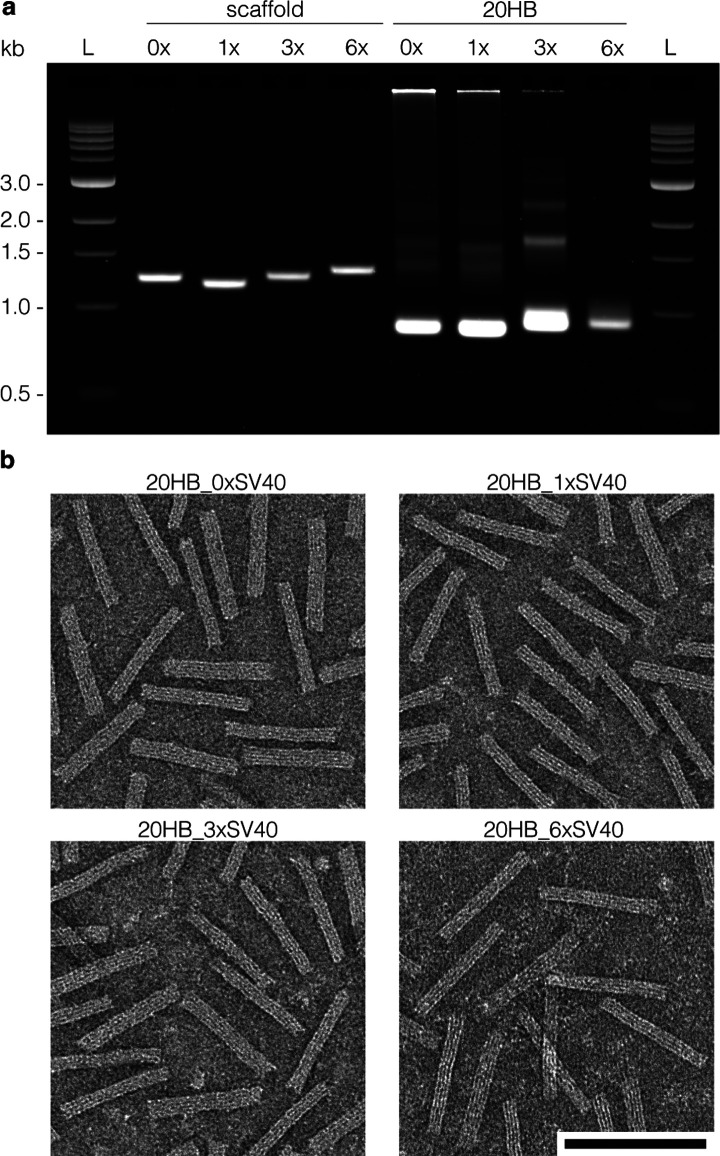
Characterization of custom scaffolds and corresponding DNA origami structures. (a) Agarose gel demonstrating all custom scaffolds produced, and the corresponding purified DNA origami structures. (b) Representative negative stain TEM images showing the 20HB DNA origami structures for each of the custom scaffolds produced. Scale bar 100 nm, ladder (L) depicts NEB 1 kb dsDNA ladder.

We were interested in delivery to dividing cells, but also in delivery to cells that were arrested at the G1/S stage in the cell cycle to limit passive nuclear uptake during mitosis. HEK293T cells were utilized in this study as they are used frequently as a model cell line for gene delivery and enable us to investigate our constructs in both dividing and arrested models of the same cell line. We tested several chemical arresting agents (Figure S4) and proceeded with aphidicolin as the arresting chemical as we achieved a stable arrest in G1/S phase. We treated the cells with 15 μM aphidicolin for 24 h prior to electroporation, and then maintained them in arresting media for the remainder of the experiment. Arrested cells appeared somewhat swollen compared to normally dividing cells (Figure 3a,b). We confirmed cell cycle arrest by flow cytometric analysis of cell cycle phases. Actively dividing cells featured a high G1 peak and a second lower G2/M phase peak separated by S phase, consistent with the literature.^20^ In contrast, the plot of the arrested cells had only one peak, demonstrating accumulation at the G1/S border and in S phase (Figure 3c, Figure S5).

**Figure 3:**
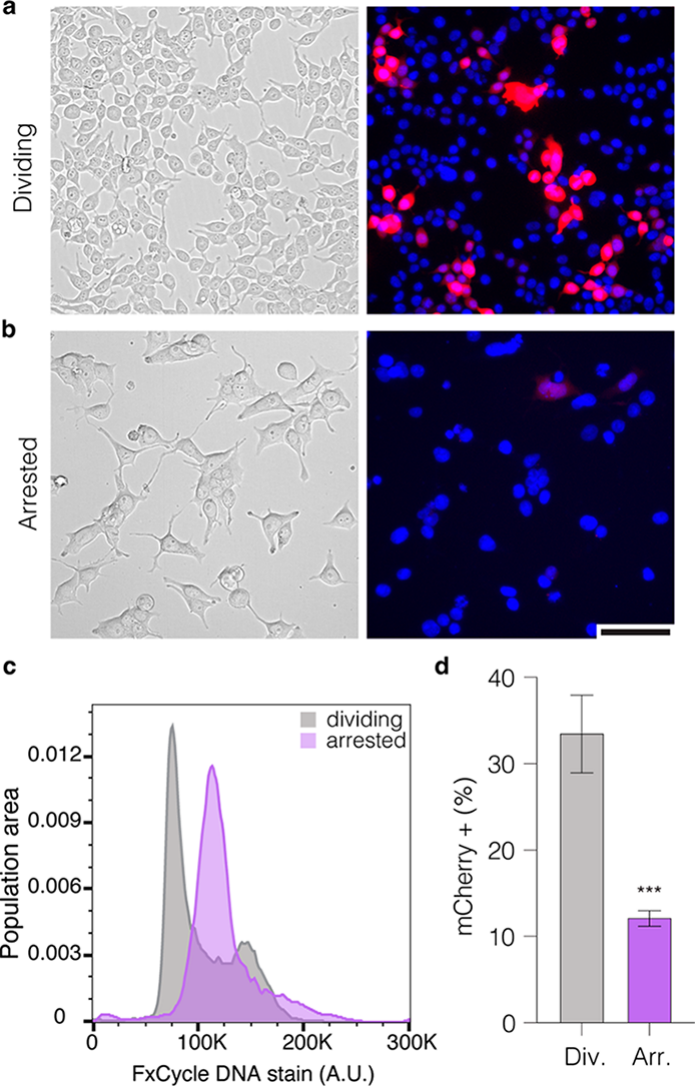
Cell cycle arrest diminishes gene delivery efficiency. Representative phase image and corresponding epifluorescence image of dividing (a) and arrested (b) HEK293T cells 24 h after electroporation with the 20HB_0×SV40 (without any SV40 sequences). mCherry signal is shown in red, nuclei in blue, scale bar 100 μm. (c) Flow cytometry histogram plot demonstrating cell cycle populations of actively dividing and chemically arrested HEK293T cells. (d) Quantification of mCherry+ cells (%) in dividing and chemically arrested HEK293T populations 24 h after electroporation with the 20HB_0×SV40. Data collected in (d) were quantified using flow cytometry and are presented as mean ± standard deviation (s.d.) for *n* = 3 biologically independent experiments. Statistical analysis for (d) was performed using Student’s *t* test (***p ≤ 0.001).

After confirming cell cycle arrest, we tested for a decrease in gene expression, as the arrest should inhibit the occurrence of passive nuclear transport during mitosis. For this purpose, we transfected both dividing and arrested cells with the 20HB_0×SV40 via electroporation. Cells were analyzed qualitatively by epifluorescence microscopy and quantified by flow cytometry. We observed a statistically significant decrease in the percentage of mCherry+ cells after electroporation in the arrested cell population (Figure 3d). The percentage of mCherry+ cells for the origami was decreased to ∼12% in arrested cells, compared to ∼34% in dividing cells. We observed a similar trend when delivering the corresponding dsDNA plasmids via lipofection (Figure S6a).

Next, we tested the 20HB variants, 20HB_0×SV40, 20HB_1×SV40, 20HB_3×SV40, and 20HB_6×SV40 in both dividing and chemically arrested HEK293T cells. We quantitatively assessed cells via flow cytometry for proportion of mCherry+ cells (%) and mean fluorescence intensity (MFI, given in arbitrary units A.U.) to signify gene expression levels. Recordings were compared to those obtained for the control, 20HB_0×SV40, that lacked the SV40 DTS (Figure 4a–e). Inclusion of the SV40 DTS sequences had a strong effect in arrested cells, where the percentage of mCherry+ cells increased for the 20HB_1×SV40 (1.4-fold), and more so for the 20HB_3×SV40 (1.8-fold), when compared to 20HB_0×SV40. The gene expression levels (MFI) showed a stronger response to the presence of the DTS, with a 3-fold increase for the 20HB_1×SV40 and 4.7-fold increase for 20HB_3×SV40 in chemically arrested cells. The structures were further screened in dividing cells, as an additional control. In dividing cells, we observed a small increase in the percentage of mCherry+ cells and in the MFI for 20HB_3×SV40. Similar trends were observed when delivering the corresponding dsDNA plasmids (Figure S6b,c). The 20HB variant with six SV40 DTS sites (20HB_6×SV40) demonstrated consistently lower proportions of mCherry+ cells and reduced MFI of mCherry expression, which we attribute as being related to the inferior folding quality of the sample, which may promote accelerated degradation of this object by nucleases.

**Figure 4:**
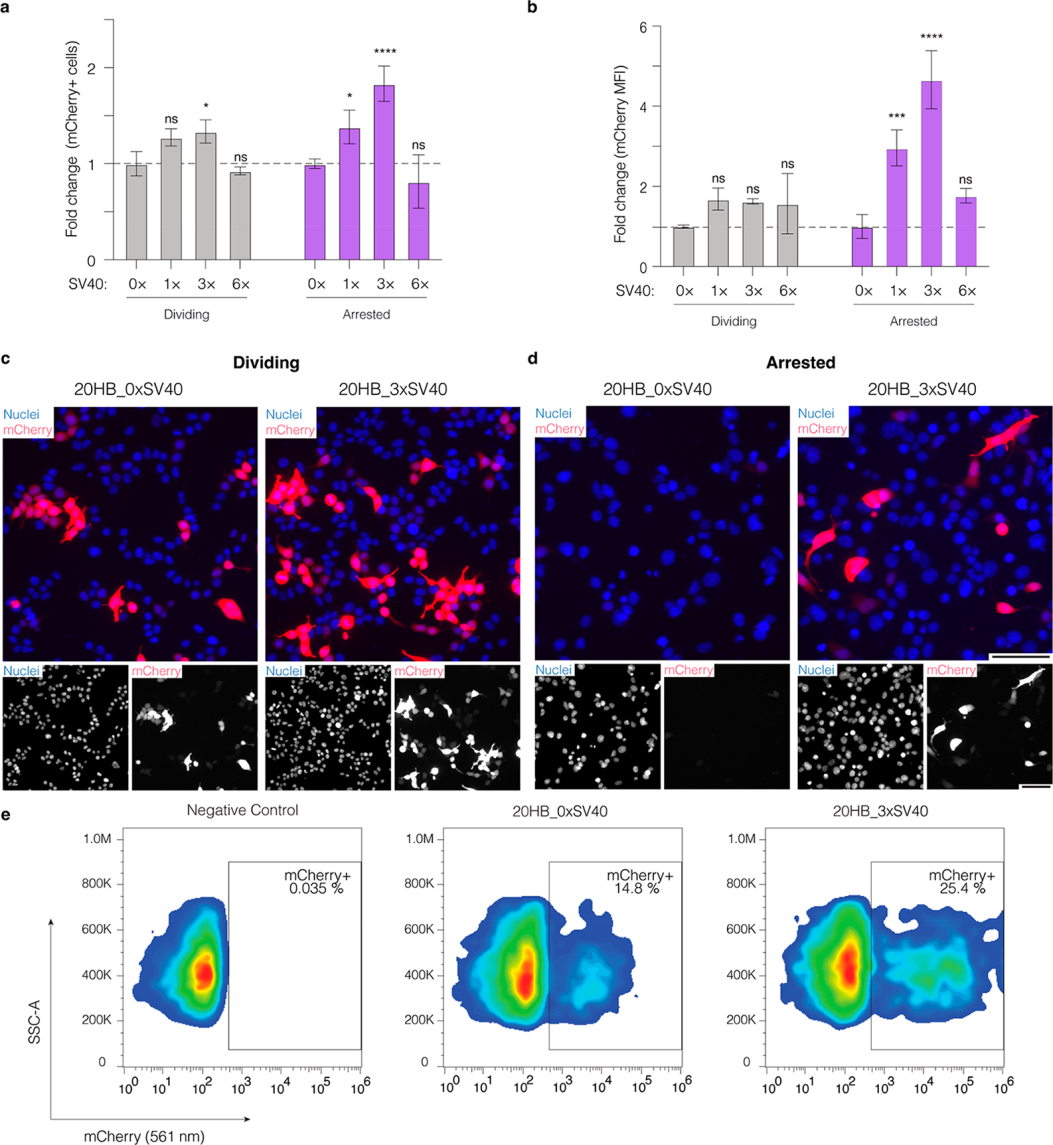
Presence of SV40 DTS sequences in DNA origami enhances gene expression through nuclear import. (a) Fold change of the percentage of mCherry+ cells and (b) mean fluorescence intensity (MFI) of mCherry in dividing and arrested cells after electroporation with 20HB variants. Both the percentage of mCherry positive cells and MFI are shown as fold change compared to the value of the control 20HB_0×SV40 in dividing and arrested cells, respectively. Data collected in (a) and (b) were quantified using flow cytometry and are presented as mean ± standard deviation (s.d.) for *n* = 3 biologically independent experiments. Statistical analysis was performed using two-way ANOVA with Dunnett’s multiple comparisons (*p ≤ 0.05, ***p ≤ 0.001, ****p ≤ 0.0001, ns *p* > 0.05). Representative epifluorescence microscopy images after electroporation of dividing cells (c) and arrested cells (d) for the control 20HB_0×SV40 and 20HB_3×SV40. Images were taken 24 h after electroporation and are representative of *n* = 3 biological replicates (similar results were observed each time); the full panel including all conditions is given in Figure S7. In overlay, mCherry signal is shown in red, nuclei are shown in blue. Scale bar is 100 μm. (e) Representative flow cytometry gates demonstrating mCherry expression (mCherry 561 nm, *x*-axis) against side scatter-area (SSC-A, *y*-axis) in chemically arrested HEK293T cells.

In conclusion, our findings show that intracellular active nuclear import of DNA origami objects can be induced and exploited by simply displaying virus-derived DTS sequences on the surface of DNA origami objects. These DTS motifs presumably recruit cellular proteins which are substrates of importin proteins involved in active transport through the nuclear pores.^21^ Future work could involve investigating various 3D origami shapes for their efficacy in enhancing binding specificity, or regulating binding interactions, together with additional sequence-encoded functions, and stability in biological media. Ultimately, utilization of DNA origami objects for gene delivery may enable development of “smart” responsive systems, multicomponent gene assemblies, and carrier-free targeted delivery. As such, our results can have important implications for biotechnological applications such as gene delivery and regulation, and intracellular biosensing based on custom DNA origami objects.

## Supporting Information Available

Experimental procedures including cloning and production of custom scaffolds, cell culture, cell arrest treatments, microscopy, and flow cytometry; Further data including information for plasmid design, chemical cell cycle arrest, 20HB characterization, electroporation, and liposomal delivery using plasmids (PDF)
