# Supplementary material for: Active Nuclear Import of Mammalian Cell-Expressible DNA Origami
Source: J Am Chem Soc. 2023 Feb 24;145(9):4946–50. doi: 10.1021/jacs.2c12733 (PMC9999407; doi:10.1021/jacs.2c12733)
Supplement: Supplementary file 1 — ja2c12733_si_001.pdf [file ja2c12733_si_001.pdf]

Active nuclear import of mammalian cell-expressible DNA origami

Anna Liedl[‡,§], Johanna Grießing[‡,§], Jessica A. Kretzmann[‡,§,†,*] and Hendrik Dietz[‡,§,*]

‡ Department of Biosciences, School of Natural Sciences, Technical University of Munich, Am Coulombwall 4a, 85748 Garching, Germany.

§ Munich Institute of Biomedical Engineering, Technical University of Munich, Boltzmannstraße 11, 85748 Garching, Germany.

Table of Contents

1. Materials & Methods 2
2. Supplemental Data 8
3. Sequences & Designs 14
  3.2 sc_mCherry_0×SV40 14
  3.2 sc_mCherry_1×SV40 17
  3.3 sc_mCherry_3×SV40 20
  3.4 sc_mCherry_6xSV40 23

1. Materials & Methods

Plasmid cloning. Plasmids encoding for custom scaffolds were created via standard cloning techniques. All plasmids were created via Golden gate assembly using BsaI-HF®v2 (NEB cat. no. R3733), together with T4 DNA ligase (NEB cat. no. M0202). For each plasmid, appropriate cut sites were introduced with PCR (Q5® High-Fidelity 2x Master Mix, NEB, as per manufacturer's protocol), and the assembly was conducted as per manufacturer's protocol. Plasmids were verified using restriction digests and DNA sequencing (Eurofins genomics, Ebersberg Germany).

Touchdown PCR with primers for all constructs are detailed in Table S1. In all cases, PCR products were confirmed by agarose gel electrophoresis (AGE), bands were excised, and fragments were extracted (Qiagen QIAquick Gel Extraction Kit) as per manufacturer's protocol. For the introduction of DTS sequences, custom sequences encoding for the 72 bp SV40 DTS and BsaI cleavage sites were ordered as ssDNA from IDT and were annealed in-house directly prior to the Golden gate assembly reaction.

Table S1 I Primer sequences used for the construction of the plasmids for mCherry-encoding custom scaffold production.

Plasmid	Template	Primer	Sequence 5' - 3'
sc_mCherry_0xSV40	Addgene plasmid #126854	FWD	CAAGGTGGTCTCCgtgacattaagcgcggcgggtg
		REV	CAAGGTGGTCTCCaatgagtgagcaaaaggccagca
	Addgene plasmid #128744	FWD	CAAGGTGGTCTCGcattcgcgatgtacgggccaga
		REV	CAAGGTGGTCTCGtcacagagccccagctggttctt
sc_mCherry_1xSV40	Addgene plasmid #126854	FWD	TTTCCGGGTCTCGgtgacattaagcgcggcgggtg
		REV	TTTCCGGGTCTCGaatgagtgagcaaaaggccagca
	sc_mCherry	FWD	TTTCCGGGTCTCCcattgtacgggccagatatacg
		REV	TTTCCGGGTCTCCATctctagactcgagcggcc
	sc_mCherry	FWD	TTTCCGGGTCTCCGCtttaaacccgctgatcagc
		REV	TTTCCGGGTCTCCtcacaggttctttccgcctcaga
	IDT SV40 seq*	S1*	TTCCGGGTCTCCAGATCCGGTGTGGAAAGTCCCCAGGCTCCCCAGCAGGCAGAAGTATGCAAAGCATGCATCTCAATTAGTCAGCAACCAAAGCTTGGAGACCAAAGGC
		S2*	CCTTTGGTCTCCAAGCTTTGGTTGCTGACTAATTGAGATGCATGCTTTGCATACTTCTGCCTGCTGGGGAGCCTGGGGACTTTCCACACCGGATCTGGAGACCCGGAAA
sc_mCherry_3xSV40_ intermediate	sc_mCherry_1xSV40	FWD	TTGTGGGGTCTCGgcctcgactgtgccttctag
		REV	TTGTGGGGTCTCGgagcggccgctcacttgtacagc
	sc_mCherry_1xSV40	FWD	TTGTGGGGTCTCGgctcgagtctagagatccg
		REV	TTGTGGGGTCTCGtttggttgctgactaattgag
	sc_mCherry_1xSV40	FWD	TTGTGGGGTCTCGcaaagctctagagatccggtgtgg
		REV	TTGTGGGGTCTCGgatggtttaaagctttggttgct
	sc_mCherry_1xSV40	FWD	TTGTGGGGTCTCGcatccggtgtggaaagtcc
		REV	TTGTGGGGTCTCGaggctgatcagcgggtttaa
sc_mCherry_3xSV40	sc_mCherry	FWD	TTCGAGGGTCTCCttgtgacattaagcgcggc
		REV	TTCGAGGGTCTCCcttacccggccctctaga
	sc_mCherry	FWD	TTCGAGGGTCTCGtaaggagggcccgtttaaaccc
		REV	TTCGAGGGTCTCGagccatagagcccaccgcat

	sc_mCherry	FWD	TTCGAGGGTCTCGggctcgctttcttgctgtcc
		REV	TTCGAGGGTCTCGcatccccagtttagtagttgg
	sc_mCherry_3xSV40_intermediate	FWD	CAAGAGGGTCTCCatgcggccgctcgagtctag
		REV	CAAGAGGGTCTCCcgaggctgatcagcgggtt
	Addgene plasmid #128744	FWD	AACACCGGTCTCGgactacaacaaggcaaggct
		REV	AACACCGGTCTCGacaaagcagcgcaaaacgcct
sc_mCherry_6xSV40	sc_mCherry_3xSV40	FWD	GTACACGGTCTCGgactatacgcgttgacattgattat
		REV	GTACACGGTCTCGccaatgagtgagcaaaaggcc
	sc_mCherry_3xSV40	FWD	GTACACGGTCTCGttggggatgcggccgctcga
		REV	GTACACGGTCTCGagtcgaggctgatcagcgg

*Sequences were annealed and used directly in the Golden gate assembly reaction, as opposed to being used as primers.

Scaffold production. The custom scaffolds were produced based on the plasmids described above. A protocol to produce custom scaffolds was presented previously[1,2] and is described in detail in the following section.

For ssDNA production, chemically competent DH5α *E. coli* cells were cotransformed with both the scaffold plasmid of interest, and a helper plasmid (Addgene, # 120346). Transformations were plated on agar plates containing 100 µg/mL carbenicillin and 50 µg/mL kanamycin. After growing overnight, a single colony was picked and grown in a 5 mL pre-culture (2xYT medium, 100 µg/mL carbenicillin, 50 µg/mL kanamycin) at 37 °C with shaking. After turning turbid (~10 h), the pre-cultures were transferred to 2.5 L Ultra Yield flasks (Thomson) containing 750 mL of 2xYT medium (50 µg/mL carbenicillin, 30 µg/mL kanamycin, 5 mM $MgCl_2$), and were grown overnight at 37 °C. 50 µL anti-foam (A8311, Sigma-Aldrich) was added per flask to avoid foam formation. The next day, the culture was transferred to 750 mL centrifuge bottles and the bacteria were removed by centrifugation at 4700*g* for 45 min at 4 °C. The supernatants were transferred to fresh centrifuge bottles where 30 g/L polyethylene glycol 8000 (PEG-8000) and 30 g/L NaCl was added and mixed for 30 min, r.t. to precipitate the phages. Afterwards, the phages were pelleted by centrifugation (1 h, 4700*g*, 4 °C), and the pellet was resuspended in 4 mL 1×TE buffer. Samples were transferred to 50 mL falcon tubes, and residual bacteria was pelleted by centrifugation (20 min, 15 000*g*, 4 °C). The supernatant was transferred to a fresh falcon tube and the single stranded DNA was extracted from the phages. First, 10 mL lysis buffer (Qiagen P2, cat. no. 19052) was added and mixed gently by inversion. Afterwards, 7.5 mL neutralization buffer (Qiagen P3, cat. no. 19053) was added and mixed by inversion. Samples were incubated on ice for 15 min, and then centrifuged (25 min, 16 000*g*, 4 °C). The supernatant was transferred to fresh falcon tubes and ethanol (22.5 mL/tube, 4 °C) was added. The falcon was incubated in an ice water bath for 30 min and centrifuged (20 min, 16 000*g*, 4 °C) to collect precipitated ssDNA. The ssDNA pellet was then washed with an additional 10 mL 75% ethanol, incubated (10 min, ice water bath), and centrifuged (20 min, 16 000*g*, 4 °C). The supernatant was carefully removed, and residual ethanol was evaporated, before the pellet was dissolved in 1-2 mL 1×TE. Scaffold concentrations were measured with a NanoDrop™8000

Spectrophotometer (Thermo Scientific) using the absorbance at 260 nm. The size and sequence of the scaffold was verified using agarose gel electrophoresis and sequencing (Eurofins genomics).

DNA origami design, folding and purification. Origami objects were designed using caDNAno v0.1 software. All origami objects were folded in standardized 'folding buffers' containing *x* mM $MgCl_2$ in addition to 5 mM Tris base, 1 mM EDTA and 5 mM NaCl, pH 8 (FOB*x*). All reactions were subjected to thermal annealing ramps in Tetrad (Bio-Rad) thermal cycling devices. Exact folding conditions for each structure is given in Supplementary Table S2 and S3. Staple strands were purchased from Integrated DNA Technologies at 100 µM with standard desalting, listed in Supplementary Tables S4–S7. Origami scaffold and staple routing are given in Fig. S7–S10. Origami objects were purified by either PEG precipitation, or gel purification, as previously described.[3,4] When necessary, DNA origami objects were concentrated *via* repetitive filter centrifugation (50 kDa Amicon ultra centrifuge filters, 10,000*g*, 2 min) as per manufacturer's protocol.

Table S2 I Folding conditions summary for each of the structures and scaffolds used.

Scaffold	Structure	Program	10× Folding Buffer	v(scaffold, 100 nM,) *µ*L*	v(staples, 100 *µ*M), *µ*L*
sc_mCherry	20HB	1	FOB10	4	8
sc_mCherry_1xSV40	20HB	1	FOB15	10	8
sc_mCherry_3xSV40	20HB	2	FOB25	4	14
sc_mCherry_6xSV40	20HB	5	FOB15	10	8

*For 20 *µ*L folding reaction, where necessary volume supplemented to 20 *µ*L total with ddH_2O.

Table S3 I Folding programs used for the folding reactions detailed in Table S2.

Program	1. Denaturation time		2. Temperature ramp	3. Storage temperature
	30 s	15 min		
**1**	-	65 °C	60–44 °C, at 1 °C/1 h	20 °C
**2**	-	65 °C	60–44 °C, at 1 °C/2 h	20 °C
**3**	70 °C	65 °C	60–35 °C, at 1 °C/1 h	20 °C
**4**	70 °C	65 °C	60–35 °C, at 1 °C/2 h	20 °C
**5**	-	65 °C	60–25 °C, at 1 °C/2 h	20 °C

Gel electrophoresis. For characterization of PCR products and plasmids, 1% agarose gels containing 0.5 × TBE buffer (22.25 mM tris base, 22.25 mM boric acid, 0.5 mM EDTA) were used. Gel electrophoresis was performed with an identical buffer solution for 1 h at a voltage of 110 V. To characterize folded origami objects and ssDNA scaffolds, we used 2% agarose gels containing 0.5 × TBE buffer and 5.5 mM $MgCl_2$. Gel electrophoresis was performed with an identical buffer solution for 1 – 2 h at a voltage of 90 V, gels were placed in a water bath for cooling. All gels were imaged using a Typhoon FLA 9500 laser scanner (GE Healthcare) with a pixel size of 50 µm/pixel.

Negative staining TEM. Samples were incubated on glow-discharged copper TEM grids (FCF400-CU, Electron Microscopy Sciences), for 30–60 s. Grids were then stained for 30 s (2% aqueous uranyl

formate, 25 mM NaOH). Imaging was performed at magnifications of 21,000–42,000×. Data was acquired with SerialEM software, using a FEI Tecnai T12 microscope (120 kV, Tietz TEMCAM-F416 camera). Images were processed using ImageJ.[5] TEM micrographs were high-pass filtered to remove long-range staining gradients and the contrast was auto-levelled using Adobe Photoshop CS5.

Cell culture. HEK293T cells (DSMZ) were cultured routinely in Dulbecco's modified Eagle's medium (DMEM, Gibco, cat. no. 31966047), supplemented with 10% heat-inactivated fetal bovine serum (FBS, Sigma-Aldrich, cat. No. F9665). Cells were grown in a humidified incubator at 37 °C with 5% CO_2, cells were routinely checked for presence of mycoplasma.

Cell cycle arrest. HEK293T cells were arrested for 24 h prior to electroporation using arrest media (DMEM supplemented 10% FBS and 5 ng/µL aphidicolin, Sigma-Aldrich, cat. no. A0781, dissolved in dimethyl sulfoxide, DMSO, Sigma-Aldrich, cat. no. D2438). Cells were kept in arresting media for the entirety of the experiment. For cell cycle arrest with thymidine the cells were treated with DMEM supplemented with 10% FBS and 2.5 mM thymidine (Sigma-Aldrich, cat. no. T1895, dissolved in ddH_2O) for 16 h, followed by 8 h without in routine growth media, and then a further 16 h of arrest. For cell cycle arrest with hydroxyurea the cells were treated with DMEM supplemented with 10% FBS and 1 mM hydroxyurea (Sigma-Aldrich, cat. no. H8627, dissolved in ddH_2O), and cells were kept in arresting media for the entirety of the experiment.

In preparation for flow cytometry, arrested and dividing cells were collected via trypsinization and fixed with 2% formaldehyde (20 min, Merck, cat. no. 1.00496.8350). Cells were centrifuged (5 min, 300*g*) and the cell pellet was washed with DPBS (Dulbecco's phosphate buffered saline, Gibco, cat. no. 14190-094), before being resuspended in DPBS with 0.1% TritonX-100, FxCycle Far Red Stain (200 nM final concentration for 1 x 10^6 cells/mL, Invitrogen cat. no. F10348) and 5 µL RNase A (Invitrogen, cat. no. 12091021), and incubated for 30 min, r.t.

Lipofection. To transfect the cells using Lipofectamine 2000 (Invitrogen, Thermo Fisher Scientific, cat. no. 11668-027) cells were seeded the day before at a density of 80,000 cells/mL in a 48-well plate either in normal or in arrest media. On the day of the transfection, plasmid was diluted to 100 ng/µL, and 5 µL of the plasmid was mixed with 25 µL of Opti-MEM (Gibco, Thermo Fisher Scientific). Next, 0.5 µL of Lipofectamine 2000 was mixed with 29.5 µL of Opti-MEM. The plasmid solution (30 µL) was thoroughly mixed into the Lipofectamine solution (30 µL) and incubated for 20 min at r.t. Then, 30 µl of the mixture was added per well to either arrested or dividing cells. After 24 h, the cells were analyzed by fluorescence microscopy and flow cytometry, as described below.

Electroporation. Electroporation experiments were carried out according to the Manufacturer's protocol (Neon™ transfection protocol, ThermoFisher). Briefly, HEK293T cells were washed with DPBS and collected using TryplE. Cells were pelleted via centrifugation (5 min, 300*g*), resuspended in DPBS

and counted. Cells were centrifuged again (5 min, 300*g*), and then resuspended in Buffer R (Neon™ Transfection System) at a concentration of 5.6 × 10^6 cells/mL. Mixtures for each condition were prepared so that each electroporation event contained 0.75 µg total DNA origami, and the volume was supplemented to a total of 1 µL with 1 × FOB5 buffer (folding buffer, 1 mM Tris, 1 mM EDTA, 5 mM NaCl, 5 mM $MgCl_2$), which was mixed with 9 µL of the cell suspension. Electroporation occurred in the 10 µL transfection tips, with one pulse at pulse voltage of 1600 V and width of 20 ms. After electroporation, cells were immediately transferred to a 48 well plate which had been pre-prepared with a poly-L-lysine coating, and 240 µL of complete DMEM growth media or arresting media. After 24 h, the cells were analyzed by fluorescence microscopy and flow cytometry, as described below.

Microscopy and flow cytometry. After 24 h, samples were imaged using the EVOS™ M7000 Imaging System, and the percentage of mCherry positive cells was quantified via flow cytometry. Briefly, samples were acquired using Attune Nxt Flow Cytometer and software (Thermo Fisher). In total, either 20,000 or 10,000 single cell events for dividing or arrest cells respectively, gated on side scatter area versus height, were recorded for analysis. mCherry was excited with a 561 nm laser and emission was measured with a 620/15 nm bandpass filter. Untreated cells, and cells electroporated with buffer only, were used as negative controls. Cell cycle arrest was confirmed by cell cycle analysis via flow cytometry. The cells were stained with FxCycle™ Far Red Stain (Invitrogen, Thermo Fisher, cat. no. F10348) according to Manufacturer's protocol (and as specified above), the dye was excited with a 638 nm laser and emission was measured with a 670/14 nm bandpass filter. Data was analyzed post-acquisition using FlowJo software (v10.7.1). Exemplary flow gating pathways are given in Fig. S8.

Statistics and reproducibility. Statistical analyses were performed with GraphPad Prism (GraphPad Software Inc. v9). The data is illustrated as the mean ± standard deviation, and the individual data points representing biological replicates are shown. The specific analysis performed is detailed in the corresponding figure caption. For all tests, differences were considered significant at $p \leq 0.05$ (*), $p \leq 0.01$ (**), $p \leq 0.001$ (***), $p \leq 0.0001$ (****).

References

(1) Engelhardt, F. A. S.; Praetorius, F.; Wachauf, C. H.; Brüggenthies, G.; Kohler, F.; Kick, B.; Kadletz, K. L.; Pham, P. N.; Behler, K. L.; Gerling, T.; Dietz, H. Custom-Size, Functional, and Durable DNA Origami with Design-Specific Scaffolds. *ACS Nano* **2019**, *13* (5), 5015–5027. https://doi.org/10.1021/acsnano.9b01025.

(2) Praetorius, F.; Kick, B.; Behler, K. L.; Honemann, M. N.; Weuster-Botz, D.; Dietz, H. Biotechnological Mass Production of DNA Origami. *Nature* **2017**, *552* (7683), 84–87. https://doi.org/10.1038/nature24650.

(3) Stahl, E.; Martin, T. G.; Praetorius, F.; Dietz, H. Facile and Scalable Preparation of Pure and Dense DNA Origami Solutions. *Angewandte Chemie* **2014**, *126* (47), 12949–12954. https://doi.org/10.1002/ange.201405991.

(4) Wagenbauer, K. F.; Engelhardt, F. A. S.; Stahl, E.; Hechtl, V. K.; Stömmer, P.; Seebacher, F.; Meregalli, L.; Ketterer, P.; Gerling, T.; Dietz, H. How We Make DNA Origami. *ChemBioChem* **2017**, *18* (19), 1873–1885. https://doi.org/10.1002/cbic.201700377.

(5) Schindelin, J.; Arganda-Carreras, I.; Frise, E.; Kaynig, V.; Longair, M.; Pietzsch, T.; Preibisch, S.; Rueden, C.; Saalfeld, S.; Schmid, B.; Tinevez, J.-Y.; White, D. J.; Hartenstein, V.; Eliceiri, K.; Tomancak, P.; Cardona, A. Fiji: An Open-Source Platform for Biological-Image Analysis. *Nat Methods* **2012**, *9* (7), 676–682. https://doi.org/10.1038/nmeth.2019.

2. Supplemental Data

a

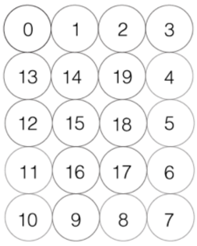

b

Structure	No. SV40 copies	Helix
20HB_0×SV40	-	-
20HB_1×SV40	1	9
20HB_3×SV40	3	10, 11
20HB_6×SV40	3	0, 1
	3	9, 10

Fig. S1 l Placement of SV40 DTS sequences in 20HB DNA origami designs. a, Schematic cross section of 20 HB, with helices labeled 0 – 19. **b**, Number of SV40 sequence repeats, and helix within the 20HB that the SV40 sequences feature.

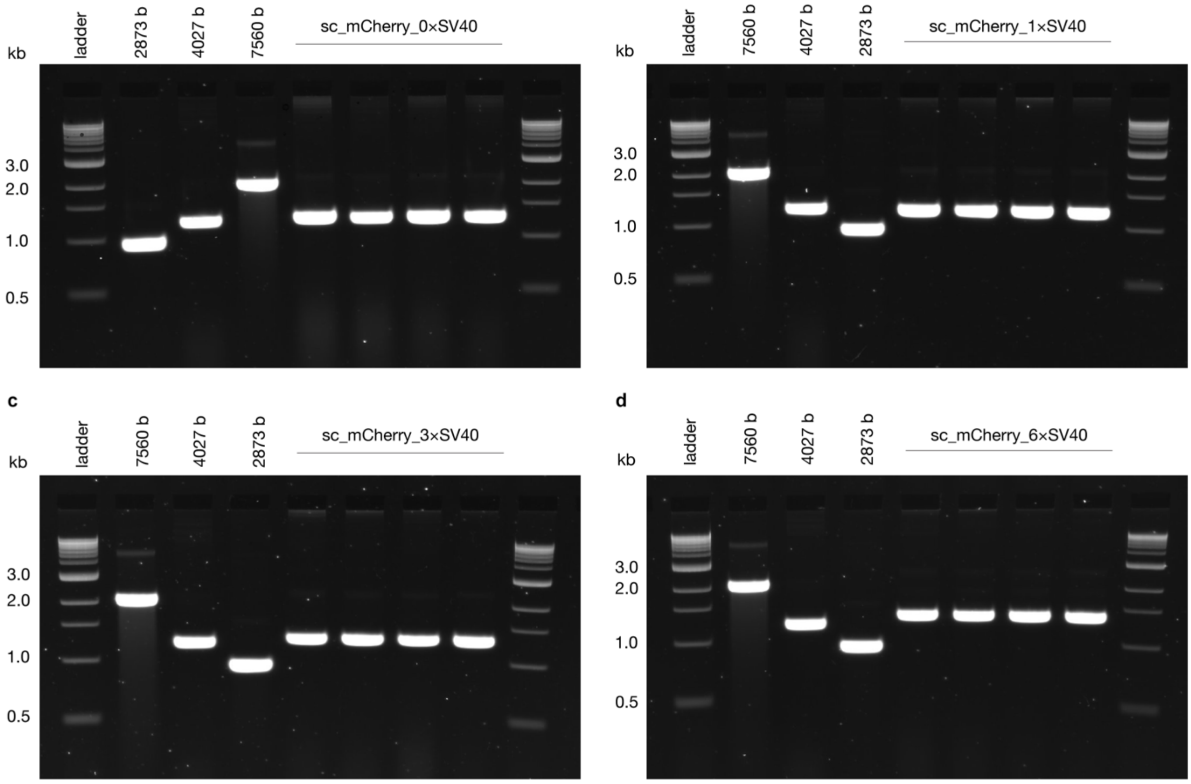

Fig. S2 l AGE showing all custom scaffolds created in this study. ssDNA markers at 2873 b, 4027 b and 7560 b were used as molecular weight markers, while 'ladder' depicts NEB 1 kb dsDNA ladder.

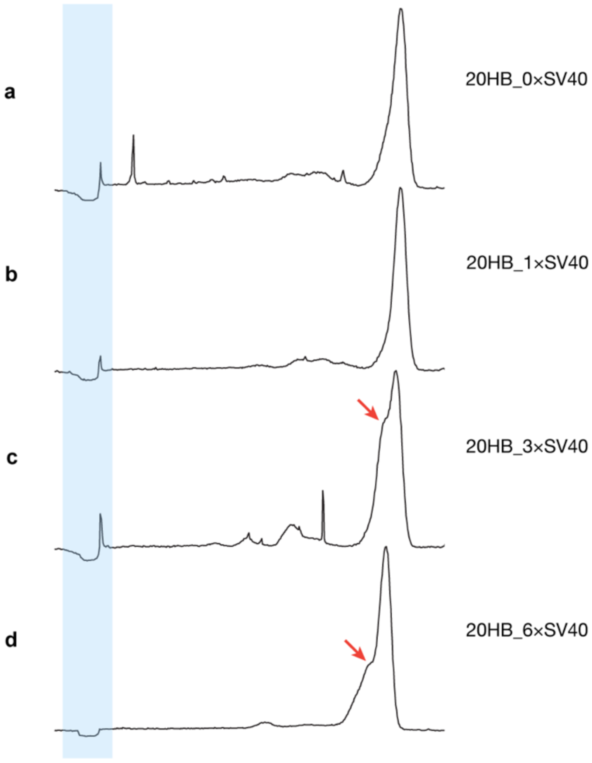

Fig. S3 I AGE lane profiles of purified DNA origami objects. Representative normalized lane profiles for purified origami objects 20HB_0×SV40 (**a**), 20HB_1×SV40 (**b**), 20HB_3×SV40 (**c**) and 20HB_6×SV40 (**d**). For reference, the well pocket of each lane is highlighted in blue, and shoulder impurities are demonstrated with an arrow (red).

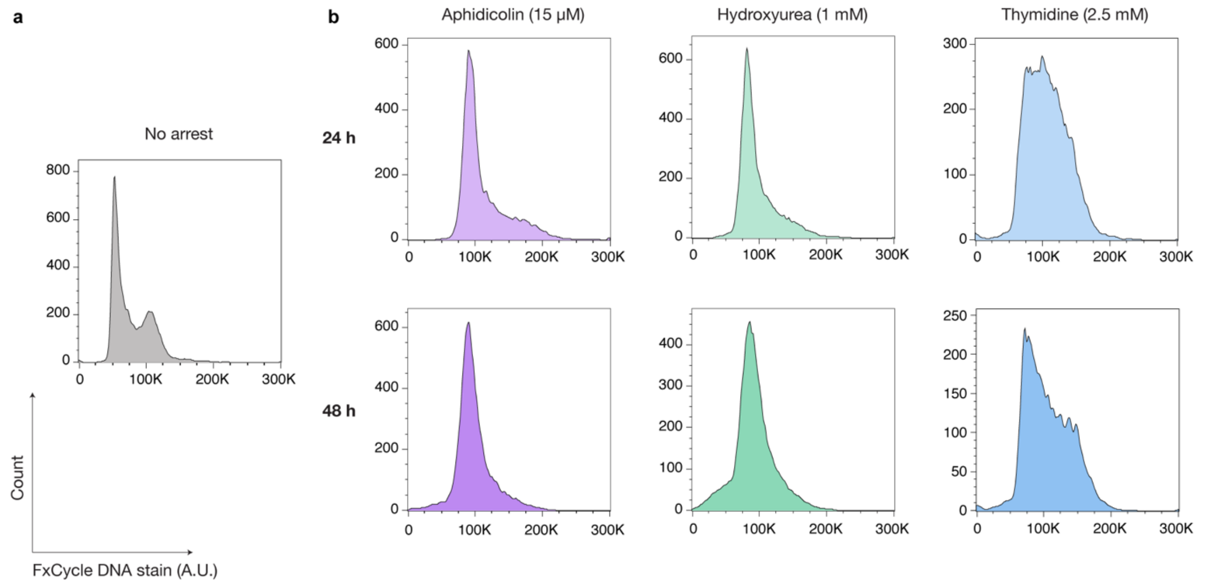

Fig. S4 I Comparison of different arresting agents. Cell cycle analysis of untreated HEK293T cells (**a**) or cells treated wither 15 μM aphidicolin, 1 mM hydroxyurea or 2.5 mM thymidine for either 24 or 48 h (**b**). Cells were analyzed by flow cytometry, and the data presented here is representative of n = 2 biologically independent experiments.

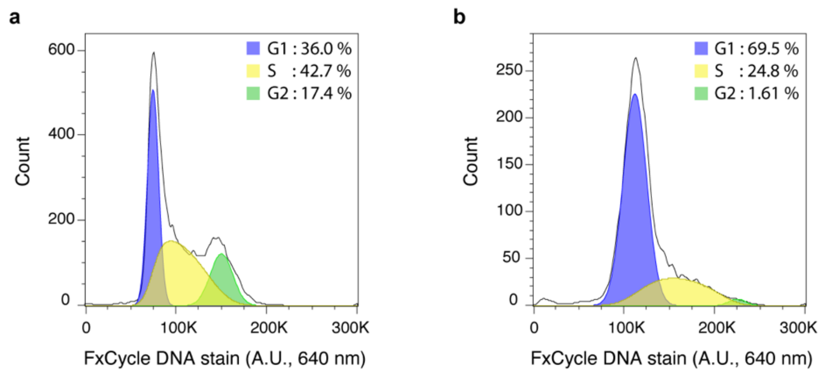

Fig. S5 I Cell cycle analysis of dividing and chemically arrested cells. Cell cycle analysis of dividing HEK293T cells (**a**) and chemically arrested HEK293T cells (**b**). Cells in G1, S and G2 phases are given in blue, yellow and green, respectively.

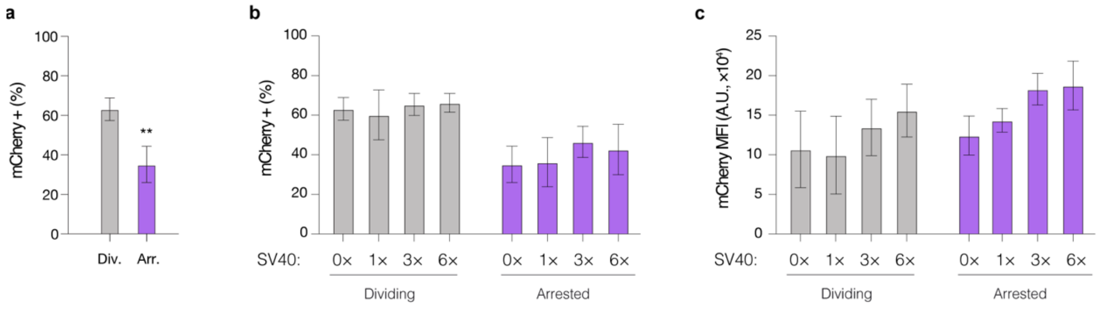

Fig. S6 I Expression of plasmid DNA in dividing and arrested cells with lipofectamine 2000 delivery. a, Quantification of mCherry+ cells (%) in dividing and chemically arrested HEK293T populations 24 h after delivery of plasmid DNA corresponding to sc_mCherry_0×SV40. **b**, Percentage of mCherry+ cells and **c**, mean fluorescent intensity (MFI) of mCherry in dividing and arrested cells after delivery of plasmid DNA variants with lipofectamine. Data for **a**–**c** collected were quantified using flow cytometry and are presented as mean ± standard deviation (s.d.) for n = 3 biologically independent experiments. Statistical analysis for **a** was performed using Student's t-test (**p ≤ 0.01), and for **b**, **c** statistical analysis was performed using two-way ANOVA with Dunnett's multiple comparisons, no significant difference was observed compared to the corresponding mCherry_0×SV40 plasmid control.

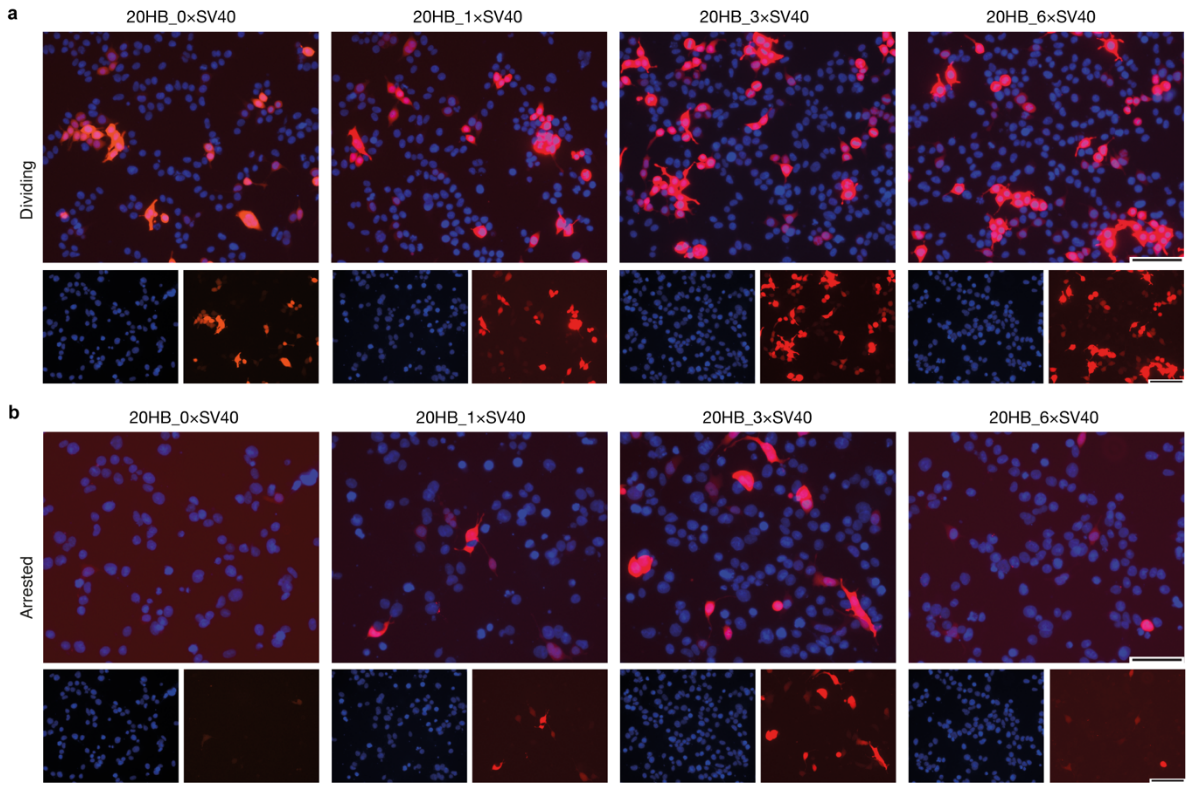

Fig. S7 l Full panel of representative images corresponding to Fig. 4. Representative fluorescence microscopy images after electroporation of dividing cells (**a**) and arrested cells (**b**) for all 20HB variants. Images were taken 24 h after electroporation and are representative of n = 3 biological replicates (similar results were observed each time). mCherry signal is shown in red, nuclei are shown in blue. Scale bar is 100 µm.

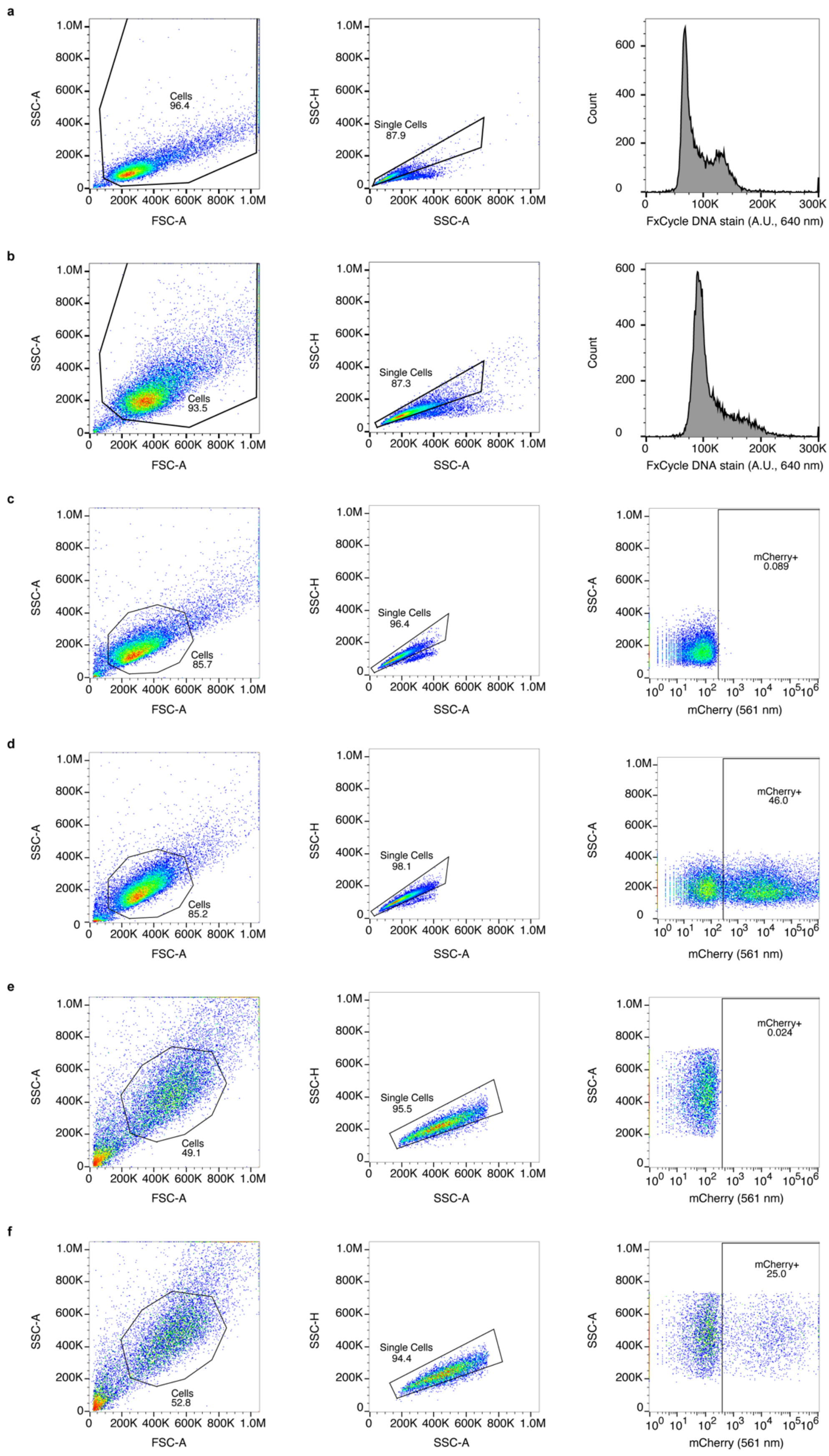

Fig. S8 I Exemplary flow cytometry gating pathways for HEK293T cells. From left to right: cell populations were first gated on forward scatter-area (FCS-A) versus side scatter-area (SSC-A), gate 'cells'; single cells were selected by gating SSC-A versus side scatter-height (SSC-H), gate 'single cells'; cells were then assessed per

specific experiment. For arrest experiments the population was plotted for FxCycle DNA stain (A.U., 640 nm excitation) versus total cell count, as demonstrated for dividing (**a**) and chemically arrested (**b**) cells. For mCherry expression experiments, cells were assessed for mCherry expression (561 nm excitation) versus SSC-A. Examples are given for dividing cells (**c** and **d**) and chemically arrested cells (**e** and **f**). Representative gating for negative controls are given in **c** and **e**.

3. Sequences & Designs

3.2 sc_mCherry_0×SV40

Sequence sc_mCherry_0×SV40:
cgcgatgtacgggccagatatacgcgttgacattgattattgactagttattaatagtaatcaattacggggtcattagttcatagcccatatatgga
gttccgcgttacataacttacggtaaatggcccgcctggctgaccgcccaacgacccccgcccattgacgtcaataatgacgtatgttcccata
gtaacgccaatagggactttccattgacgtcaatgggtggagtatttacggtaaactgcccacttggcagtacatcaagtgtatcatatgccaagt
acgccccctattgacgtcaatgacggtaaatggcccgcctggcattatgcccagtacatgaccttatgggactttcctacttggcagtacatctac
gtattagtcatcgctattaccatggtgatgcggttttggcagtacatcaatgggcgtggatagcggtttgactcacggggatttccaagtctccacc
ccattgacgtcaatgggagtttgttttggcaccaaaatcaacgggactttccaaaatgtcgtaacaactccgccccattgacgcaaatgggcggt
aggcgtgtacggtgggaggtctatataagcagagctctctggctaactagagaacccactgcttactggcttatcgaaattaatacgactcacta
tagggagacccaagctggctagcgtttaaacttaagcttggtaccgagctcggatccactagtccagtgtggtggaattcgccaccatggtgag
caagggcgaggaggataacatggccatcatcaaggagttcatgcgcttcaaggtgcacatggagggctccgtgaacggccacgagttcga
gatcgagggcgagggcgagggccgcccctacgagggcacccagaccgccaagctgaaggtgaccaagggtggcccctgcccttcgcc
tgggacatcctgtcccctcagttcatgtacggctccaaggcctacgtgaagcaccccgccgacatccccgactacttgaagctgtccttccccg
agggcttcaagtgggagcgcgtgatgaacttcgaggacggcggcgtggtgaccgtgacccaggactcctccctgcaggacggcgagttcat
ctacaaggtgaagctgcgcggcaccaacttcccctccgacggccccgtaatgcagaagaagaccatgggctgggaggcctcctccgagcg
gatgtaccccgaggacggcgccctgaagggcgagatcaagcagaggctgaagctgaaggacggcggccactacgacgctgaggtcaa
gaccacctacaaggccaagaagcccgtgcagctgcccggcgcctacaacgtcaacatcaagttggacatcacctcccacaacgaggact
acaccatcgtggaacagtacgaacgcgccgagggccgccactccaccggcggcatggacgagctgtacaagtgagcggccgctcgagtc
tagagggccgggtaagctcgctttcttgctgtccaatttctattaaaggttcctttgttccctaagtccaactactaaactggggattcctgggccctg
aagaagggcccctcgactaagtccaactactaaactgggccctgaagaagggcccatatagggccctgaagaagggccctatcgaggata
ttatctcgactaagtccaactactaaactgggccctgaagaagggcccatatagggccctgaagaagggccctatcgaggatattatctcgag
gatattatgaagggccttgagcatctggattctattctatagtgtcacctgctagagggcccgtttaaacccgctgatcagcctcgactgtgccttct
agttgccagccatctgttgtttgcccctcccccgtgccttccttgaccctggaaggtgccactcccactgtcctttcctaataaaatgaggaaattgc
atcgcattgtctgagtaggtgtcattctattctggggggtggggtggggcaggacagcaagggggaggattgggaagacaatagcaggcatg
ctggggatgcggtgggctctatggcttctgaggcggaaagaaccagctggggctctgtgacattaagcgcggcgggtgtggtggttacgcgca
gcgtgaccgctacacttgccagcgccctagcgcccgctcctttcgctttcttcccttcctttctcgccacgttcgccggctttccccgtcaagctctaa
atcgggggctccctttagggttccgatttagtgctttacggcacctcgaccccaaaaaacttgattagggtgatggttcacgtagtgggccatcgc
cctgatagacggtttttcgccctttgacgttggagtccacgttctttaatagtggactcttgttccaaactggaacaacactcaaccctatctcggtct
attcttttgatttataagggattttgccgatttcggcctattggttaaaaaatgagctgatttaacaaaaatttaacgcgaattttaacaaaatattaac
gcttacaatttaggtggcacttttcggggaaatgtgcgcggaacccctatttgtttatttttctaaatacattcaaatatgtatccgctcatgagacaat
aaccctgataaatgcttcaataatattgaaaaaggaagagtatgagtattcaacatttccgtgtcgcccttattcccttttttgcggcattttgccttcct
gtttttgctcacccagaaacgctggtgaaagtaaaagatgctgaagatcagttgggtgcacgagtgggttacatcgaactggatctcaacagc
ggtaagatccttgagagttttcgccccgaagaacgttttccaatgatgagcacttttaaagttctgctatgtggcgcggtattatcccgtattgacgc
cgggcaagagcaactcggtcgccgcatacactattctcagaatgacttggttgagtactcaccagtcacagaaaagcatcttacggatggcat
gacagtaagagaattatgcagtgctgccataaccatgagtgataacactgcggccaacttacttctgacaacgatcggaggaccgaaggag
ctaaccgcttttttgcacaacatgggggatcatgtaactcgccttgatcgttgggaaccggagctgaatgaagccataccaaacgacgagcgt
gacaccacgatgcctgtagcaatggcaacaacgttgcgcaaactattaactggcgaactacttactctagcttcccggcaacaattaatagact
ggatggaggcggataaagttgcaggaccacttctgcgctcggcccttccggctggctggtttattgctgataaatctggagccggtgagcgtgg
gtctcgcggtatcattgcagcactggggccagatggtaagccctcccgtatcgtagttatctacacgacggggagtcaggcaactatggatga
acgaaatagacagatcgctgagataggtgcctcactgattaagcattggtaactgtcagaccaagtttactcatatatactttagattgatttaaaa
cttcatttttaatttaaaaggatctaggtgaagatcctttttgataatctcatgaccaaaatcccttaacgtgagttttcgttccactgagcgtcagacc
ccgtagaaaagatcaaaggatcttcttgagatcctttttttctgcgcgtaatctgctgcttgcaaacaaaaaaaccaccgctaccagcggtggttt
gtttgccggatcaagagctaccaactctttttccgaaggtaactggcttcagcagagcgcagataccaaatactgtccttctagtgtagccgtagtt
aggccaccacttcaagaactctgtagcaccgcctacatacctcgctctgctaatcctgttaccagtggctgctgccagtggcgataagtcgtgtct
taccgggttggactcaagacgatagttaccggataaggcgcagcggtcgggctgaacggggggttcgtgcacacagcccagcttggagcg
aacgacctacaccgaactgagatacctacagcgtgagctatgagaaagcgccacgcttcccgaagggagaaaggcggacaggtatccgg
taagcggcagggtcggaacaggagagcgcacgagggagcttccagggggaaacgcctggtatctttatagtcctgtcgggtttcgccacctc
tgacttgagcgtcgatttttgtgatgctcgtcaggggggcggagcctatggaaaaacgccagcaacgcggcctttttacggttcctggccttttgct
ggccttttgctcactcatt

Corresponding 20HB DNA origami design and staple list for sc_mCherry_0×SV40:

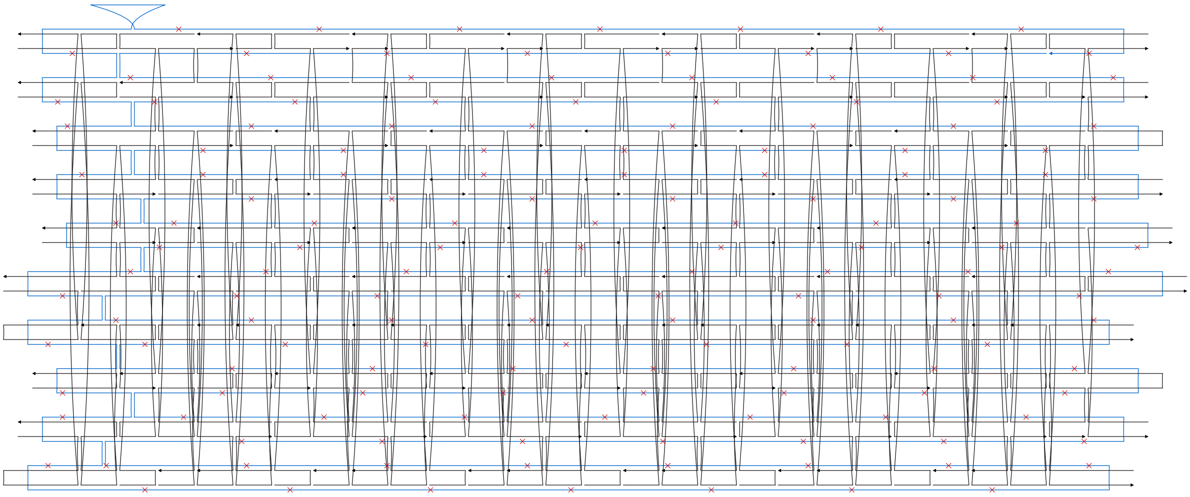

Fig. S9 l Scaffold routing and staple design for 20HB_0×SV40. Scaffold routing is shown in blue, staples are given in black, skips are given as red crosses. Design was prepared using caDNAno v0.1.

Table S4 l Individual staple sequences for sc_mCherry 20HB.

Name	Sequence 5' - 3'
core_1	CCGCTTACCGGATCCAACATACGTATTTAGA
core_2	CTCCCTCGTGCGCTCGGGTCGTTATTGTCTC
core_3	TATAAAGATACCAGGTTACCGTATTTCAATA
core_4	TCAAGTCAGAGGTGGATATGGGCACGGAAAT
core_5	CCCCCCTGACGAGCAGATTACTAAAAATGCC
core_6	CCGCGTTGCTGGCGTTGTCAACGCAGCGTTTC
core_7	TGGGCGGTCCTGTTCCGCACATTTCCCCGATAGAGCTTGTCATTCT
core_8	CGGGCCATCGTTTCCCTGTAAGCGTTAATATTAAATCGGAGTTGCTC
core_9	AACTCCATCGAAACCCTTAAATTTTTGTTAAAGTTTTTTATACCGCG
core_10	CCGTAATTTCACAAAAACCAATAGGCCGAAATGGCCCACGCTCATCA
core_11	TAATCAATTTTCCATATAAATCAAAAGAATATCAAAGGGAAACTCT
core_12	CGTCAATGATGGAAAAAACCACCATGAGCGGTCAACCAAGACGGGG
core_13	GGAAAGTCTTTACCGTTGCAAGCTTATTGAAGGCGACCGAACCCTA
core_14	AACTCCCTGATACAGGATCTCGTTGAATAACGGGATAGGGGTCGA
core_15	TGGAAATATTGACGTCGGGGTCGCAAAAAATTAAAAGTTACGTGAA
core_16	CGCCCATGGGCATAACACGTTAATGGGTGAGCGGGGCGACGAAAAAC
core_17	ACCATGGTCAAGTAGGAAAAAGGACTTCAGCAGTTGAGATATTAAAGA
core_18	AGCTCTGAACTGAGGAGGGCTTCAGATTT
core_19	AGTAAGCGCAGGGGGCTCCCCGTGAAGGGCC
core_20	CTATAGTGGGCGGTCTCTGTCTATTTTATCCG
core_21	TAAGTTTATCGCCCTCGCTTAATCTGCCGGGA
core_22	GGACTAGTCGTTCACGTATGAGTATTAATAGT
core_23	CTCGCCCTTGCTCACCGCGCATGAAAATGAATACAGGCA
core_24	TCGGGGATACTTGATACTGTTCCACGATGGT
core_25	GAAGCCCTCTGCACGCCGGTGGAGTGGCGGC
core_26	CGTCCTCGCTTGACCTGGCCGCTCACTTGTAC
core_27	GAGGAGTCTTCAGCTTGCGAGCTTACCCGGC
core_28	CACCTTGTTCAGGGCGGAACCTTTAATAGAA
core_29	TGGGAGGTGATGTCCAGTCGGCGACCGGCTCACCATCTG
core_30	TGTAGGCGCCGGGCAGCGGGGAAGCCAGCCGCGTGTAG
core_31	GGCCTTGTAGGTGGTAAGTTCATCTGCAACTTCGTTC
core_32	TAGTGGCCGCCGTCCCTGGGTCATAATTGTAGTGAGG
core_33	GCTTGATCTCGCCCTAGATGAACTTCGCCAGAACTTGGT
core_34	GGGGTACATCCGCTCGGGAAGTTGCCATTGCGTTTTAAA
core_35	CAAGGCCGTAGTCCCCACGCTCGGGTGCTTTTGGAGCCGTACATG

core_36	ATCCTCGACCTCGGCTAAACCAGGACAGCTTGTCCCAGGCGAAGG
core_37	TATGGGCCAGCTCGTCAAGTGGTCCACGCGCGGTCACCTTCAGCTT
core_38	GTTGGACTCCTCTAGACAGTCTATCGGTCACTCGTAGGGGCGGCCC
core_39	GGGCCCTTATTGGACATAAGTAGTCGCCGTCATCTCGAACTCGTGGC
core_40	TCTTCAGGGACTTAGGCGTTGTTGGTGCCGCCCATGTGCACCTTGAA
core_41	AGAATCCAGCGAAAGGCCGTAAGGTGACTGGGCCCCAGT
core_42	ACGGGCCCGTGTAGCGGCACTGCGAGAATAGATAACTAC
core_43	AGGCACAGACCCGCCGGGCCGCAGTTGCCCGATCCATAG
core_44	AGGGGCAACTGGTTCTCGGTCCTCCACATACACCTATC
core_45	GCACCTTCCCACCGCCCCATGTTTTGGAAACTGACAG
core_46	TCATTTTTTCCCAATTCCCAACCAAGGATCTCAATCT
core_47	CGAACGTGACAAATAGTTTGAATGTCATTATTTGGCGTTACTATGGG
core_48	CCCCGATTAAAGTGCCTCAGGGTTGGGCGGTC
core_49	TAAAGCACTTTGTTAATCTTCCTTAGTTATGT
core_50	CCTAATCAATCAGCTCGGGCGACTATGAACT
core_51	AGGGCGATCGGCAAAGGAAGGCTTAATAA
core_52	TCCAACGGACCGAGATTTCACCGTATATC
core_53	TGAGTACATACATAGGGTTCCGCGACCCTG
core_54	TGTATGCGCATTTAACCTAAATCCTGGAAG
core_55	GCGTCAATCTCATACAATTCGCGGACAGGAC
core_56	GCAGAACTGGGAATAAATTTTTTAATCGACGC
core_57	ACGTTCTTCAAAAACAATCCCTTAGGCTCCG
core_58	TTACCGCTTCTTTTACTAGGGTTTAAAAAGG
core_59	CCACCCATTGACGTCAGGGCGGAAGCCAGAG
core_60	CATAAGGTCATGTACTTGATGTACACCACACT
core_61	AGTGGGTTTTTTTTGTTAAATACTAGCCAGG
core_62	GGATCCGAGAAAACTTGCCAGGCTAGTCAA
core_63	ATAATTCTATCAGCAAGCGTTCGTGTTGACGT
core_64	ACTCATGGTTCTTCAGGATCAGCGGGTTTAA
core_65	CGAGTTACCCTATAAGGACAGTGGGAGTG
core_66	GATCAAGGTTGCGCAAGAACAAAGCCGTCCTC
core_67	ACTGCCAAGTGGGCAGCCGTTGAGATAAGCC
core_68	GGGCGTACTTGGCATAATTGACGTGGGTCTCC
core_69	CGGGCCATTTACCGTCCCCCGTGAACCAAGCT
core_70	CTTATATACGGCAAACGTCCCTATGACGTCAA
core_71	AGTCGTAGAAAAAAACTTGATGTAACGCGG
core_72	AACGCTACTTTTCTACAATAGGAATGACC
core_73	ATGGTGGCGATTATCAAAGTCCTGGCCCGT
core_74	ATGCTTACCGCGAGACTCGACTATCGGTATTG
core_75	CTTACTGTTATCCTCGGTGACACTATAGAAT
core_76	TGTTATCGAGCGCAGCATGCCGGGCTTCTT
core_77	TTGTCAGAGGGCCCATGGCTGGCAACTAGA
core_78	CCGATCGCCTCCATCCTCGAGCCAGCGTCG
core_79	AAAGCGGTATAATATAGGAAGGCACGGGGG
core_80	GTGCAAAAGCTAGAGGCAAGAAACAGCCTCT
core_81	TTCATTCTTAGTAGTATGCGATGCAATTTCC
core_82	CTTCATAACATGCCATAGCGGGCGAAAGCCGG
core_83	TAGGGCCCTTATGGCAGTCACGCAAGGGAGC
core_84	CTTCTTCAAGTAAGTTCGCTTAAGGTGCCG
core_85	TAGTCGAGTAGCTCCTTTTCCGCCCCATCAC
core_86	CTTCAGGGCATGATCCATCCCCAGCGTCTATC
core_87	GCCCAGTAGCTCCGGTCCTCCCCACGTGGAC
core_88	CAGGAAAGGGAAGAAAGATCGACAGACAGCT
core_89	CTAGGGCGCTGGCAATCTAGCAGAGATAAT
core_90	TGCGCGTAACCACCACTCGAGGCTGGCCCTA
core_91	TGTCACAGAGCCCCAGACAACAGAGTTTAGTA
core_92	TCAGAAGCCATAGAGCCAGGGTCACCTCGATA
core_93	CATGCCTGCTATTGTCATTAGGAATGGGCCCT
core_94	TCTTGATCGACCTCCCGCCTACCGCCCATTTG
core_95	GCTGGTAGATACGGGGGACAGGATCAAGTAG
core_96	GCGGTGGTCTCTAGTTGTTGTTACGACATTTT

core_97	AGCAGATTTGCCTGACCACCCTTTCCCACTT
core_98	TACGCGCATTAATTTCTTTTGGTGCCAAAACA
core_99	AAGAAGATTCAGCGATGGGTGCCCCACGCCGC
core_100	CCTTTGATGCCAGCTTCAATGGGGTGGAGACT
core_101	TGACGCTCTTACCAATGCCCTCGCTGCAGG
core_102	AGTGGAACGCTCGGTGTCAAACCGCTATCCA
core_103	GGGATTTTAAAGTATAGAGCCCTGCAGCTT
core_104	GGTCATGAGAATTCCTGCCAAAACCGCATC
polyT_1	TTTTTGGCCAGCAAAAGGCCAAATGAGTGAACTGATTCTTCACCTAGATTTTTT
polyT_2	TTTTTGCTTTCTCATAGCTCCTTCGGGAGCTACAGAAAAATAAGCGAGAAAGGCTTTCT
polyT_3	ACATCGCGGGAACCGGAGTGTTGTTCCAGTTTGGAATTTTT
polyT_4	TTTTTACGTAGATGTACTGCAATAGCGA
polyT_5	TTTTTAGTTCGGTGTAGGTCACGCTGTAAAAAGAGTGTTCTTGAAGTGGTTTTT
polyT_6	GTTACACACCGTCGCTCCAAGCTGGGCTTTTT
polyT_7	TTTTTTGTGTGCACGAACGACTCTGCTGCGTACACTAGAAGGACAGTATTTTT
polyT_8	TTTTTTCTTCTGCATTGATGATGGCCATTTTTTTTTTTGTTATCCTCTGACTAATTTTTT
polyT_9	TTTTTGCTGCGCCTTATCCGTCTTGAGTTTTTT
polyT_10	AGGAATCCCCTCCCAGCCCATGGTCTTTTTT
polyT_11	TTTTTTTCTTCAGGGCCCGTCACGCTTTTTT
polyT_12	TTTTTCCAACCCGGTATTATCGCCACTGGCAGCAGTTTTT
polyT_13	TTTTTATAGAATGACACCTACACCCCACCCCCCAGATTTTT
polyT_14	TTTTTCCACTGGTAAAGAGCGAGGTATTTTTTTTTTTGTAGGCGGTAGCGTGGCTTTTT
polyT_15	CTTGCTGTCCTGCCCCTCAGACATGGACTTAGTCGAGGGGCCCTTTTT
polyT_16	TTTTTCAAGAGTCCACTCCAGTTCGGTATGGCTCGTGGT
polyT_17	TTTTTTGGCCTAACTACGGTTAGC
polyT_18	TTTTTCGTCGTTTGATGTAACCCATTTTTTTTTTCTCGTGCACCCAGCAAAATTTTT
polyT_19	TTTTTTTTGGTATCTGAAGCCAGTTTTTTTTTTTTACCTTCGGAGGTATCTCTTTTT
polyT_20	TTTTTCCTTTTAAATTAAACTCCTTACGGGGCCGTCGGAGGGAGGAGGCCAGTTTAGTAG TTG
polyT_21	TGGTAGCGCTGCAATCCCAGGCCCACGTCCGTTCAGCCCGACCTTTTT

3.2 sc_mCherry_1×SV40

Sequence sc_mCherry_1×SV40:
gtacgggccagatatacgcgttgacattgattattgactagttattaatagtaatcaattacggggtcattagttcatagcccatatatggagttccg
cgttacataacttacggtaaatggcccgcctggctgaccgcccaacgacccccgcccattgacgtcaataatgacgtatgttcccatagtaacg
ccaatagggactttccattgacgtcaatgggtggagtatttacggtaaactgcccacttggcagtacatcaagtgtatcatatgccaagtacgcc
ccctattgacgtcaatgacggtaaatggcccgcctggcattatgcccagtacatgaccttatgggactttcctacttggcagtacatctacgtatta
gtcatcgctattaccatggtgatgcggttttggcagtacatcaatgggcgtggatagcggtttgactcacggggatttccaagtctccaccccattg
acgtcaatgggagtttgttttggcaccaaaatcaacgggactttccaaaatgtcgtaacaactccgccccattgacgcaaatgggcggtaggc
gtgtacggtgggaggtctatataagcagagctctctggctaactagagaacccactgcttactggcttatcgaaattaatacgactcactatagg
gagacccaagctggctagcgtttaaacttaagcttggtaccgagctcggatccactagtccagtgtggtggaattcgccaccatggtgagcaa
gggcgaggaggataacatggccatcatcaaggagttcatgcgcttcaaggtgcacatggagggctccgtgaacggccacgagttcgagatc
gagggcgagggcgagggccgcccctacgagggcacccagaccgccaagctgaaggtgaccaagggtggcccccctgcccttcgcctggg
acatcctgtcccctcagttcatgtacggctccaaggcctacgtgaagcaccccgccgacatccccgactacttgaagctgtccttccccgaggg
cttcaagtgggagcgcgtgatgaacttcgaggacggcggcgtggtgaccgtgacccaggactcctccctgcaggacggcgagttcatctaca
aggtgaagctgcgcggcaccaacttcccctccgacggccccgtaatgcagaagaagaccatgggctgggaggcctcctccgagcggatgt
accccgaggacggcgccctgaagggcgagatcaagcagaggctgaagctgaaggacggcggccactacgacgctgaggtcaagacca
cctacaaggccaagaagcccgtgcagctgcccggcgcctacaacgtcaacatcaagttggacatcacctcccacaacgaggactacacca
tcgtggaacagtacgaacgcgccgagggccgccactccaccggcggcatggacgagctgtacaagtgagcggccgctcgagtctagagA
TCCGGTGTGGAAAGTCCCCAGGCTCCCCAGCAGGCAGAAGTATGCAAAGCATGCATCTCAATTA
GTCAGCAACCAAAGCtttaaacccgctgatcagcctcgactgtgccttctagttgccagccatctgttgtttgcccctcccccgtgccttcc
ttgaccctggaaggtgccactcccactgtcctttcctaataaaatgaggaaattgcatcgcattgtctgagtaggtgtcattctattctggggggtgg
ggtggggcaggacagcaagggggaggattgggaagacaatagcaggcatgctggggatgcggtgggctctatggcttctgaggcggaaa
gaacctgtgacattaagcgcggcgggtgtggtggttacgcgcagcgtgaccgctacacttgccagcgccctagcgcccgctcctttcgctttctt
cccttcctttctcgccacgttcgccggctttccccgtcaagctctaaatcgggggctccctttagggttccgatttagtgctttacggcacctcgaccc
caaaaaacttgattagggtgatggttcacgtagtgggccatcgccctgatagacggtttttcgccctttgacgttggagtccacgttctttaatagtg
gactcttgttccaaactggaacaacactcaaccctatctcggtctattcttttgatttataagggattttgccgatttcggcctattggttaaaaaatga

gctgatttaacaaaaatttaacgcgaattttaacaaaatattaacgcttacaatttaggtggcacttttcggggaaatgtgcgcggaacccctattt
gtttatttttctaaatacattcaaatatgtatccgctcatgagacaataaccctgataaatgcttcaataatattgaaaaaggaagagtatgagtattc
aacatttccgtgtcgcccttattcccttttttgcggcattttgccttcctgtttttgctcacccagaaacgctggtgaaagtaaaagatgctgaagatca
gttgggtgcacgagtgggttacatcgaactggatctcaacagcggtaagatccttgagagttttcgccccgaagaacgttttccaatgatgagca
cttttaaagttctgctatgtggcgcggtattatcccgtattgacgccgggcaagagcaactcggtcgccgcatacactattctcagaatgacttggtt
gagtactcaccagtcacagaaaagcatcttacggatggcatgacagtaagagaattatgcagtgctgccataaccatgagtgataacactgc
ggccaacttacttctgacaacgatcggaggaccgaaggagctaaccgcttttttgcacaacatgggggatcatgtaactcgccttgatcgttgg
gaaccggagctgaatgaagccataccaaacgacgagcgtgacaccacgatgcctgtagcaatggcaacaacgttgcgcaaactattaact
ggcgaactacttactctagcttcccggcaacaattaatagactggatggaggcggataaagttgcaggaccacttctgcgctcggcccttccgg
ctggctggtttattgctgataaatctggagccggtgagcgtgggtctcgcggtatcattgcagcactggggccagatggtaagccctcccgtatc
gtagttatctacacgacggggagtcaggcaactatggatgaacgaaatagacagatcgctgagataggtgcctcactgattaagcattggtaa
ctgtcagaccaagtttactcatatatactttagattgatttaaaacttcatttttaatttaaaaggatctaggtgaagatcctttttgataatctcatgacc
aaaatcccttaacgtgagttttcgttccactgagcgtcagaccccgtagaaaagatcaaaggatcttcttgagatcctttttttctgcgcgtaatctgc
tgcttgcaaacaaaaaaaccaccgctaccagcggtggtttgtttgccggatcaagagctaccaactctttttccgaaggtaactggcttcagcag
agcgcagataccaaatactgtccttctagtgtagccgtagttaggccaccacttcaagaactctgtagcaccgcctacatacctcgctctgctaat
cctgttaccagtggctgctgccagtggcgataagtcgtgtcttaccgggttggactcaagacgatagttaccggataaggcgcagcggtcggg
ctgaacggggggttcgtgcacacagcccagcttggagcgaacgacctacaccgaactgagatacctacagcgtgagctatgagaaagcgc
cacgcttcccgaagggagaaaggcggacaggtatccggtaagcggcagggtcggaacaggagagcgcacgagggagcttccaggggg
aaacgcctggtatctttatagtcctgtcgggtttcgccacctctgacttgagcgtcgatttttgtgatgctcgtcaggggggcggagcctatggaaa
aacgccagcaacgcggcctttttacggttcctggccttttgctggccttttgctcactcatt

Corresponding 20HB DNA origami design and staple list for sc_mCherry_1×SV40:

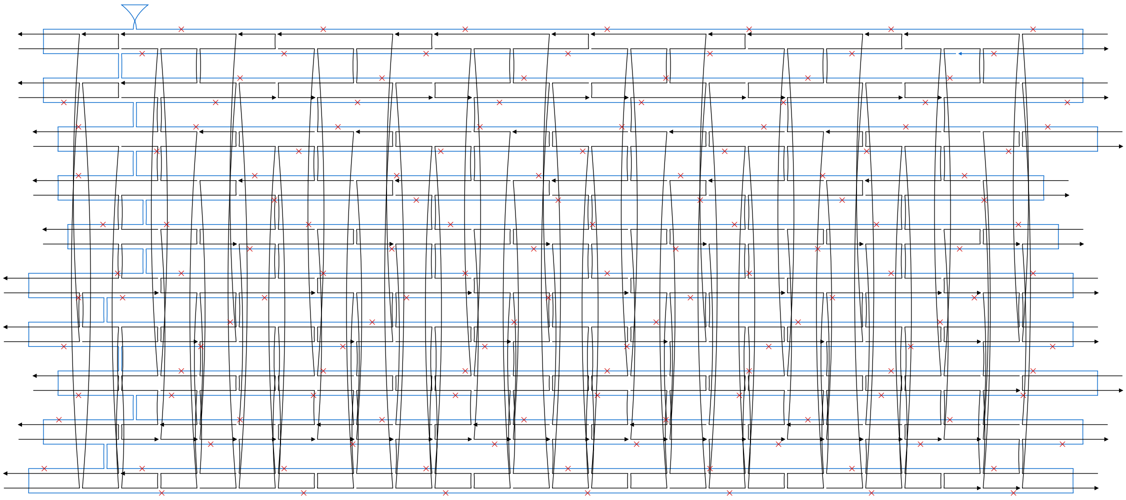

Fig. S10 l Scaffold routing and staple design for 20HB_1×SV40. Scaffold routing is shown in blue, staples are given in black, skips are given as red crosses. Design was prepared using caDNAno v0.1.

Table S5 l Individual staple sequences for sc_mCherry_1×SV40 20HB.

Name	Sequence 5' - 3'
core_1	TGACGTCACTCTTCCGAAAAAGATACGGGA
core_2	CAGCCAGTAAGGGCGACAAACCGCCTGACGGTCTGGACTGAGGGGACAGGA
core_3	GTAACGCGCAGGAAGGTTTGCAAGCGATCTCCCTCGCTGCTTCACGTAGGCC
core_4	CTAATGACACTTTCAAAAGGATCACCAATGCTTCACGGACAGCTTCAAGTAGTCG
core_5	ACTAGTCATCGTGCACTACGGGGAGTATATAGCATGAACCGCGCTCCCACTTGAA
core_6	CTGGCCCGCGCTGTTGCTCACGTTAAATTAAACTCCTCGCGTCACCACGCCGCCGT
core_7	TTGGCGTTCCGGCAAACACGGAACTCTTACTCGTGAACCAACCCTAAAGGGAGC
core_8	TCCACCCAGTTTTTTTGCAAAATGTTCTGTGAAAAAACCGGACGGGGAAAGCCGG
core_9	TACTGCCACAGAAAACCAGCGTTTTCTGAGATAAAGAACAGGAAGGGAAGAAAGC
core_10	GGGGCGTAATCTTTTCCCAACTGGCTCTTGGTTGTTCCTAGGGCGCTGGCAAGT
core_11	GCGGGCCAACGAAAAAGATCCACGCGCCAATCAAAAGCGCGTAACCACCACAC
core_12	TACACGCCTACCGCCCAGCTTGGCTCCCCGTCTGATACCG
core_13	AGAGAGCTCTGCTTATGGCCCTCGGTCTATTAGATTTAT

core_14	GCCAGTAAGCAGTGGGTGGCCGTTAATCAAGGGCCGA
core_15	TCCCTATAGTGAGTCTTGAAGCTGAGTAATATCCGCC
core_16	GCTTAAGTTTAAACGTGTTATCAATGAAGTCCGGGAAG
core_17	GCGAAGGGACCCTTGGTCACCTTCATTTGCGT
core_18	GTACATGAGTGCCCTCGTAGGGGCATAGACCT
core_19	CGGCGGGGCCTCGATCTCGAACTCGTTCTCTA
core_20	GGGAAGGAGCCCTCCATGTGCACCGTATTAAT
core_21	TTCATCATCCTTGATGATGGCCACTAGCCAG
core_22	GGTCACGCCTTGCTCACCATGGCCGAGCTCTTGGTCA
core_23	TCAGCCTAGGTGGTCTTGACCTCCGGGGGAG
core_24	GCCGTCCCCGGGCAGCTGCACGGACTAGAA
core_25	GCCTCCCGATGTCCAACTTGATGGTTTAAA
core_26	ACGGGGCCCACGATGGTGTAGTCTGCATGC
core_27	GCAGCTTCAGTGGCGGCCCTCGGGAGCCTGG
core_28	TAGTTATCGCGCCTGGCCGCCGTCCTTCAGCTTGTCCCAGGCTGCAA
core_29	GCCTTGTCTGCTTGATCTCGCCCTTCAGGGCTTGGAGCCCCGGCTCC
core_30	GTAGGCGTCGGGGTACATCCGCTCGGAGGAGGGGATGTCAGCCGGA
core_31	GGGAGGTAGCCCATGGTCTTCTTCTGCATTGCCCTCGTGCAACTT
core_32	TACTGTTCGTCGGAGGGGAAGTTGGTGCCGCCCTCGAAGAATTGTTG
core_33	GGTCGGTACAACCCAAGGAAGGCAAGCGTCGGCCCCAGT
core_34	AGGAAAGGCAACAGATGGCTGGCAGCTTCTTGACGCTCA
core_35	GACAATGCGAGGCTGATCAGCGGTTGACGTTAACCAGC
core_36	CCACCCCTGCTGACTAATTGAGACTCGTTGTGTGGTCC
core_37	CCAATCCCTTCTGCCTGCTGGGCGCGTTCGAGTCTATT
core_38	CCGCATCCCACACCGGATCTCTATCCATGCCAAGTAGTT
core_39	TGTAGGCGTTTTTCAATTTTTTGGGCCTATGGATGGTTAAACCATCTG
core_40	TATCAGGGTTATTGTCGCCCACTAGTCATGCCAGAAGTACGAGACCC
core_41	TATTTGAATGTATTTAAAAGGGCGCTGGTGACGGTTAGCCAGCAATA
core_42	AGGGGTTCCGCGCACTCCACTATATAGTGTGTTACATGGCGCAGAA
core_43	CCACCTAAATTGTAAGGTTGAGTCCCGGCGTATTCAGCTTCCATCC
core_44	AAAATTCGCGTTAAACTTATAACATAGCAGTGGTGTCACTAGAGT
core_45	TACTCATAATGGGCGGAACATACGTCATTAT
core_46	AAGGGAAGCGGGCCACAATGGAAAGTCCCTATGATTTTG
core_47	AGCAAAAAGAACTCCAAGTTTACCGTAAATACCGTCAATG
core_48	CATCTTTTCCCGTAATATGATACACTTGATGTGAGTCAA
core_49	TAACCCACATAATCAACATTGACGTCAATAGTACTGCC
core_50	GATCTTACTACAATGATGGGCATAATGCCAGCGATGAC
core_51	CTCTCAAGAAATCAGCGTAAAAAGGCCGCGTTGCTGGCGT
core_52	CAGCACTAACTACGAGTTGGTACAATGGGTACGACATTTTGGAAAGTCCCGT
core_53	CATCCGTACCATAGTTACCGCTGGCCCACCGGTGCCAAAACAAACTCCCATTGA
core_54	GTACTCAACCTATCTCAGCAGCAGGTTAGCCGGGTGGAGACTTGGAAATCCCCG
core_55	ATGCGGCGTGACAGTTTCAAGAAGTTCGATAAACCGCTATCCACGCCCATTGATG
core_56	CAATACGGCAATCTAATCTGACGCCTTGGGTCAAAACCGCATCACCATGGTAATAG
core_57	CGCAGTGTTAAATCGGATCACCCTAATCAAGTATTATTG
core_58	AGTTGGCGGGCAAAACAGTGGGAGTGGCACCTTCCAGTAAAGCACTATCACTC
core_59	TCCTCCGTAGAGCTTTCTATCAGGGCGATGTCATGAG
core_60	TCCTTCGGGGCACAGTCGATGCAATTTCCTCATTTTATTCCCCGATTATCGTTGT
core_61	TGTTGTGGCGAGAAGTGGACTCCAACGTCGAAAAAT
core_62	ATCCCCCAGCTTTGGTCCAGAATAGAATGACACCTACTCACGAACGTGCAAAAAAG
core_63	CAACGATCCGGGCGCAGTTTGGAACAAGAGATTTCCCC
core_64	CCGGTTCCTTTGCATATCCCCCTTGCTGTCCTGCCCCACCGAAAGGAGAAGGCGA
core_65	GTTTGGTACACGCTGAATAGACCGAGATAGGCGTTAAT
core_66	CGCTCGTCGGACTTTCCCAGCATGCCTGCTATTGTCTTCGTAGCGGTTGGCTTC
core_67	GGCATCGAACTTTAAATCCTTTTAAGGGATT
core_68	TTGCTACAGCTTAATGGAAATCGGCAAAATCCTTTTTGTT
core_69	GTGTAGATGCATAATTATGTTGAAAAGCATTCCGACCCTGCCGCTTACCGGGTC
core_70	TCGTTCATAGATGCTTCCGCAAAACGGATACACCCTGGAAGCTCCCTCGTGCGCT
core_71	GTGAGGCACCAAGTCATCTGGGTGAAACAAATCGACAGGACTATAAAGATACCAG
core_72	ACTTGGTCACCGAGTTATCTTCAGGAAAAGTGAATCGACGCTCAAGTCAGAGGTG
core_73	TTTAAATGATAATACGTTCGATGATTTTGTTTAGGCTCCGCCCCCCTGACGAGC
core_74	GCTCTTGATACTATGGGGGGTCGTTGGGCGGTCTCCTGTT

core_75	TAGCGGTGTTGACGTTTTACCGTAAGTTATGCGTTTCC
core_76	ATTACGCGAGTGGGCTATATGGGCTATGAAGCGAAACC
core_77	ATCCTTTGCTTGGCATTGATTACTATTAATAATCACAAA
core_78	TCAGTGGATTTACCGTTGTCAACGCGTATATTTTTCCA
polyT_1	TTTTTAGGCCAGGAACCTCATTTTTTATTTTT
polyT_2	TTTTTGGAAGCGTGGCGCTTTCGCCTTTC
polyT_3	TCATGTACGTGAGCAAAAGGCCAGCAAATTTTT
polyT_4	TTTTTAAGTCCCATAAGGTGAGATTATCATTTTT
polyT_5	TTTTTTAGGTATCTCAGTTCCTCATAGCTACCTTCGGTGCTACAGAGTTTTTTT
polyT_6	GGTGTTGTGCGGAGTAGGTCGTTCGCTCTTTTT
polyT_7	GGTACCAATAATACGTAGATGTACTGCCAAGTAGGATTTTT
polyT_8	TTTTTACTGGACTAGTGGATTGGCGAATTCCACCACTTTTT
polyT_9	TTTTTCAAGCTGGGCTGTGGGGGTATCTGCGCTCTGTTTTT
polyT_10	GTGGGCCCAGGGCACGAACCCCCCGTTTTTTT
polyT_11	TTTTTGGGAGGAGTCCTGCGCCAGTTCACCTAGAAGTGCTC
polyT_12	TTTTTCAGCCCGACCGCTCGGTAACTATTTTTT
polyT_13	GCCGGTGGACCTTGTAGATGAACTCGCCGTCCTGCATTTTT
polyT_14	TTTTTCACTTGTACAGCTCGGACTCGAG
polyT_15	TTTTTCGTCTTGAGTCAGACACGACTTATCGCCATTTTT
polyT_16	TTTTTTTCCGCCTCAGAAGCCATAGAGCCCACCGCCGC
polyT_17	TTTTTCTGGCAGCAGCAGGATTAGCATTTTT
polyT_18	TTTTTACCAATAGGCCTCACAGGTTCTTTTTT
polyT_19	TTTTTGAGCGAGGTATCCCTTCGTTTTT
polyT_20	TTTTTTCTTCGGGGCGAAAAATCATTGGAAAACGTTTTTT
polyT_21	TTTTTCTTGAAGTGGTGGGTGGTAACCACTCGAGGTGCCG
polyT_22	TTTTTAACGTTGTTGCCACGGCCGCTTTTTT
polyT_23	TTTTTAAGGACAGTATTTCTTCTACGGCTACACTAGTTTTT
polyT_24	TTTTTAAAAGGATCTTAATAGTTTGCGCTTTTT
polyT_25	TTTTTCTGAAGCCAGTTCACGCTGTTTTT

3.3 sc_mCherry_3×SV40

Sequence sc_mCherry_3×SV40:
cgcgatgtacgggccagatatacgcgttgacattgattattgactagttattaatagtaatcaattacggggtcattagttcatagcccatatatgga
gttccgcgttacataacttacggtaaatggcccgcctggctgaccgcccaacgacccccgcccattgacgtcaataatgacgtatgttcccata
gtaacgccaatagggactttccattgacgtcaatgggtggagtatttacggtaaactgcccacttggcagtacatcaagtgtatcatatgccaagt
acgccccctattgacgtcaatgacggtaaatggcccgcctggcattatgcccagtacatgaccttatgggactttcctacttggcagtacatctac
gtattagtcatcgctattaccatggtgatgcggttttggcagtacatcaatgggcgtggatagcggtttgactcacggggatttccaagtctccacc
ccattgacgtcaatgggagtttgttttggcaccaaaatcaacgggactttccaaaatgtcgtaacaactccgccccattgacgcaaatgggcggt
aggcgtgtacggtgggaggtctatataagcagagctctctggctaactagagaacccactgcttactggcttatcgaaattaatacgactcacta
tagggagacccaagctggctagcgtttaaacttaagcttggtaccgagctcggatccactagtccagtgtggtggaattcgccaccatggtgag
caagggcgaggaggataacatggccatcatcaaggagttcatgcgcttcaaggtgcacatggagggctccgtgaacggccacgagttcga
gatcgagggcgagggcgagggccgcccctacgagggcacccagaccgccaagctgaaggtgaccaagggtggcccctgcccttcgcc
tgggacatcctgtcccctcagttcatgtacggctccaaggcctacgtgaagcaccccgccgacatccccgactacttgaagctgtccttccccg
agggcttcaagtgggagcgcgtgatgaacttcgaggacggcggcgtggtgaccgtgacccaggactcctccctgcaggacggcgagttcat
ctacaaggtgaagctgcgcggcaccaacttcccctccgacggccccgtaatgcagaagaagaccatgggctgggaggcctcctccgagcg
gatgtaccccgaggacggcgccctgaagggcgagatcaagcagaggctgaagctgaaggacggcggccactacgacgctgaggtcaa
gaccacctacaaggccaagaagcccgtgcagctgcccggcgcctacaacgtcaacatcaagttggacatcacctcccacaacgaggact
acaccatcgtggaacagtacgaacgcgccgagggccgccactccaccggcggcatggacgagctgtacaagtgagcggccgctcgagtc
tagagggccgggtaaggagggcccgtttaaacccgctgatcagcctcgactgtgccttctagttgccagccatctgttgtttgcccctcccccgtg
ccttccttgaccctggaaggtgccactcccactgtcctttcctaataaaatgaggaaattgcatcgcattgtctgagtaggtgtcattctattctgggg
ggtggggtggggcaggacagcaagggggaggattgggaagacaatagcaggcatgctggggatgcggtgggctctatggctcgctttcttg
ctgtccaatttctattaaaggttcctttgttccctaagtccaactactaaactggggatgcggccgctcgagtctagagATCCGGTGTGGA
AAGTCCCCAGGCTCCCCAGCAGGCAGAAGTATGCAAAGCATGCATCTCAATTAGTCAGCAACCA
AAGCtctagagATCCGGTGTGGAAAGTCCCCAGGCTCCCCAGCAGGCAGAAGTATGCAAAGCATGC
ATCTCAATTAGTCAGCAACCAAAGCtttaaaccATCCGGTGTGGAAAGTCCCCAGGCTCCCCAGCAG
GCAGAAGTATGCAAAGCATGCATCTCAATTAGTCAGCAACCAAAGCtttaaacccgctgatcagcctcgactac
aacaaggcaaggcttgaccgacaattgcatgaagaatctgcttagggttaggcgttttgcgctgcttgtgacattaagcgcggcgggtgtggtg

gttacgcgcagcgtgaccgctacacttgccagcgccctagcgcccgctcctttcgctttcttcccttcctttctcgccacgttcgccggctttccccgt
caagctctaaatcgggggctccctttagggttccgatttagtgctttacggcacctcgaccccaaaaaacttgattagggtgatggttcacgtagt
gggccatcgccctgatagacggtttttcgccctttgacgttggagtccacgttctttaatagtggactcttgttccaaactggaacaacactcaacc
ctatctcggtctattcttttgatttataagggattttgccgatttcggcctattggttaaaaaatgagctgatttaacaaaaatttaacgcgaattttaaca
aaatattaacgcttacaatttaggtggcacttttcggggaaatgtgcgcggaacccctatttgtttatttttctaaatacattcaaatatgtatccgctca
tgagacaataaccctgataaatgcttcaataatattgaaaaaggaagagtatgagtattcaacatttccgtgtcgcccttattcccttttttgcggcat
tttgccttcctgtttttgctcacccagaaacgctggtgaaagtaaaagatgctgaagatcagttgggtgcacgagtgggttacatcgaactggatct
caacagcggtaagatccttgagagttttcgccccgaagaacgttttccaatgatgagcacttttaaagttctgctatgtggcgcggtattatcccgt
attgacgccgggcaagagcaactcggtcgccgcatacactattctcagaatgacttggttgagtactcaccagtcacagaaaagcatcttacg
gatggcatgacagtaagagaattatgcagtgctgccataaccatgagtgataacactgcggccaacttacttctgacaacgatcggaggacc
gaaggagctaaccgcttttttgcacaacatgggggatcatgtaactcgccttgatcgttgggaaccggagctgaatgaagccataccaaacga
cgagcgtgacaccacgatgcctgtagcaatggcaacaacgttgcgcaaactattaactggcgaactacttactctagcttcccggcaacaatt
aatagactggatggaggcggataaagttgcaggaccacttctgcgctcggcccttccggctggctggtttattgctgataaatctggagccggtg
agcgtgggtctcgcggtatcattgcagcactggggccagatggtaagccctcccgtatcgtagttatctacacgacggggagtcaggcaactat
ggatgaacgaaatagacagatcgctgagataggtgcctcactgattaagcattggtaactgtcagaccaagtttactcatatatactttagattga
tttaaaacttcatttttaatttaaaaggatctaggtgaagatcctttttgataatctcatgaccaaaatcccttaacgtgagttttcgttccactgagcgtc
agaccccgtagaaaagatcaaaggatcttcttgagatcctttttttctgcgcgtaatctgctgcttgcaaacaaaaaaaccaccgctaccagcgg
tggtttgtttgccggatcaagagctaccaactctttttccgaaggtaactggcttcagcagagcgcagataccaaatactgtccttctagtgtagcc
gtagttaggccaccacttcaagaactctgtagcaccgcctacatacctcgctctgctaatcctgttaccagtggctgctgccagtggcgataagtc
gtgtcttaccgggttggactcaagacgatagttaccggataaggcgcagcggtcgggctgaacggggggttcgtgcacacagcccagcttgg
agcgaacgacctacaccgaactgagatacctacagcgtgagctatgagaaagcgccacgcttcccgaagggagaaaggcggacaggta
tccggtaagcggcagggtcggaacaggagagcgcacgagggagcttccagggggaaacgcctggtatctttatagtcctgtcgggtttcgcc
acctctgacttgagcgtcgatttttgtgatgctcgtcaggggggcggagcctatggaaaaacgccagcaacgcggcctttttacggttcctggcct
tttgctggccttttgctcactcatt

Corresponding 20HB DNA origami design and staple list for sc_mCherry_3×SV40:

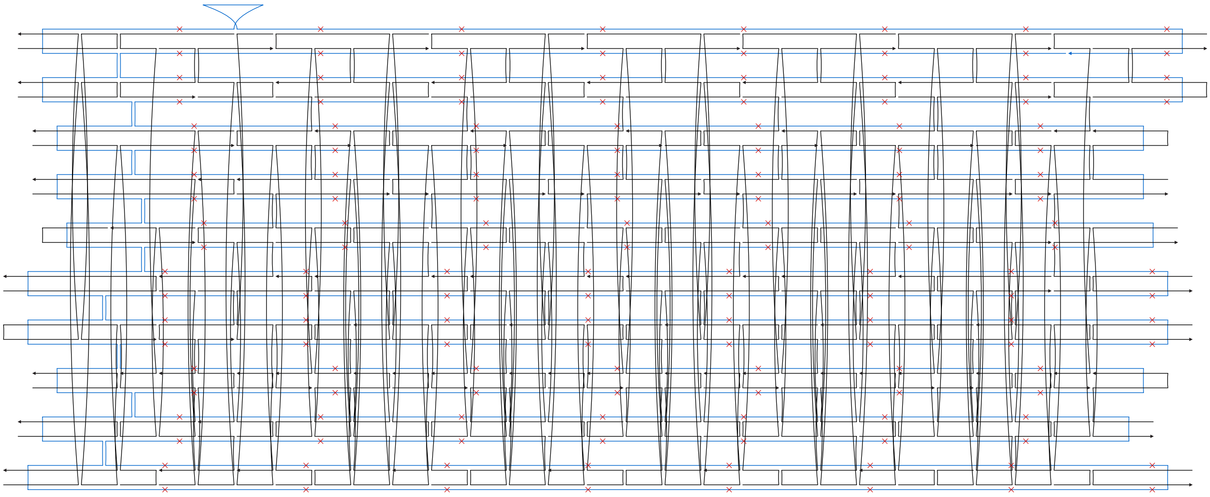

Fig. S11 l Scaffold routing and staple design for 20HB_3×SV40. Scaffold routing is shown in blue, staples are given in black, skips are given as red crosses. Design was prepared using caDNAno v0.1.

Table S6 l Individual staple sequences for sc_mCherry_3×SV40 20HB.

Name	Sequence 5' - 3'
core_1	CTTTCTCCCTTCGGGAAGCGTGGGGAACATAAAGTGCCA
core_2	GCTTACCGTTGTTAAAATTCGCGCTAGGGCCGTCAATA
core_3	CCCTCGTGTCAGCTCATTTTTTAAAGGAAGCAGAACTT
core_4	TAAAGATACGGCAAAATCCCTTATTGACGGCGTTCTT
core_5	AAGTCAGAACCGAGATAGGGTTGGGAACCCTTACCGCT
core_6	CCCTGACGGAACAAGAGTCCACTTGGGGTCCACTCGT
core_7	CGTTGCTACTCCAACGTCAAAGGACTACGTGTTTTACTT
core_8	CGTCATTATTGGCGTTACTATGCTGTGCTTATATAGA
core_9	GTGAGCAAAATACGTAGATGTACTTGCCAAAATGATGATG

core_10	GCCAAGTTAAATTAAGAAGGCACAGCATCAACCATCACTGGGGAC
core_11	CCTCCCACCGTACACGTCAATGGATTTGTTTGGGTTCCGC
core_12	CCTACCGCAGTAAGCAGTGGGTTCCGTAGGCCACGCTCAC
core_13	CCATTTGCGTCAATGGGGCGGAGCAGTTTACAAAAAGGATTGAATGT
core_14	TTGTTACCTATAGTGAGTCGTATGGACAGGAGCCCCAGT
core_15	GACATTTTGGAAAGTCCCGTTGATATATGATATTCTACGGCAGGGTTA
core_16	TTTGGTGAAGTTTAAACGCTAGCCACCCTTATAACTAC
core_17	CCAAAACAAACTCCCATTGACGTCGTCATTGAAACTCACCTTCCTTT
core_18	AATGGGGGACTAGTGGATCCGAGGGTGCCCTCCATAG
core_19	TGGAGACTTGGAAATCCCCGTGACTGGGCATATCAAAGGGCGACA
core_20	GTCAAACCGCTCACCATGGTGGCGCCCTCGAACCTATC
core_21	TCTAGTTAGTGGTTTTAAGTCCCTATTGACGTCAATGGGC
core_22	GCCATGTGAGCCCTCTGACAGTGCTCCGGCCATGTTATTTCCTC
core_23	AACTGAGGTAATTTCGGCGCAGAACGTAAATAGTCAGCCAGGCGGGCC
core_24	GCAGGGGGCCAGCTTGTTGATCTTCACTTGATTGTAACGCGGAACTCC
core_25	GGCGGTCTGCTCGGTAGGAACGAACGTCAATAACTAATGACCCCGTAA
core_26	TCGCCCTCGAATTCCACATGAGATTAATGCCAAACTAGTCAATAATC
core_27	CGTTCACGTATCCTCCATCCTTTAGGAAAGTTCTGGCCCGTACATC
core_28	CACGCGCTCCTCCATCGCTGCAATATACCGCGATTTAGAA
core_29	CGGTCACCAGCTAGAGGATACGGGGCTCATCATTGTCTCA
core_30	TCGCCGTCTTGCGCAATTGCCTGAAAACTCTCTTCAATAT
core_31	GGTGCCGCTCGTGGTGTCAGCGATCCAGTTCGCGGAAATG
core_32	TCTGCATTTTCATTCATACCAATCTGATCTTAAATGCCG
core_33	CCTCGTTGGTGGCGGCCCTCGGCAACAAAGAGTCATTC
core_34	ACTTGTACAGCTCGTCGCAAGAAATGCTTTTCCTAAGCAGGCGAAAGG
core_35	TACCCGGCCCTCTAGACCCCAGCATAATTCTCAGCCTTGCGGCGAACG
core_36	GGCTGATCAGCGGGTTCTCCCCCTTGTTATCAGCGGGTTTGCCCCCGA
core_37	CAGATGGCTGGCAACTCCCAGAATCCGATCGTGAGATGCACGTAAAGC
core_38	GTCAAGGAAGGCACGGGCGATGCAGTGCAAAAGGGGAGCCCCTAAT
core_39	AATAGAAATTGGACACATGCCGTTATCCGCCCACTTGTACATG
core_40	TAGAGCCCACCGCATCTCGAGCTGCCGGGAACGCCGCGCGAAGG
core_41	ATTGTCTTCCCAATCTAAACGGTTAATAGTCTGCAGGGTTCAGCTT
core_42	TGCCCCACCCCACCCAGAAGGCATACAGGCAGCAGCTTCGGCGGCCC
core_43	ACCTACTCAGACAATGGGAGGGGGGTATGGCACGGGGCCCTCGTGGC
core_44	TGCATGCTTTGCATACTTCTGCCTGCCTGCTAAGCGGTTGCACCCAA
core_45	CGCGCTTTGAGAATAGTGTATCCCAGCCGGACTCCCGGTA
core_46	AATGTCACGTGTAGCGGTCACGCGTAAGCGCCCCGAA
core_47	GCCTAACCTGTGACTCGGGATAGATACCGCCAGATTACATAAGCC
core_48	GTCGGTCATTACTGTCTAAAAGTAGGGCTTAAAGATCCTGGTCTCC
core_49	GCTGATCACTCATGGTCGGGGCGACTCCCCGTCGCTCAGTCCAAGCTT
core_50	GACTAATTTGTCAGAAGTTGAGATCTGTCTATATTTTGGTCCACACTG
core_51	TGCGCGTAGGTATGCGGCGACCGGATTTAT
core_52	AGCGGGCGTTAAATTTAAATAAATTGGGCGCTCCACCCATTGACG
core_53	TGGCGAGAACCAATAGTGAGCGGGTAAGTTAGTACTGCCAAGTGGG
core_54	TTTAGAGCTAAATCAATATTGAAGGGCTATGAGGGGGCGTACTTGGC
core_55	ACTAAATCAGTGTTGTTTGAATACCTATTAATGGCGGGCCATTTACC
core_56	CAAGTTTTTATTAAAGCAAAAAAGCGCGTATACCCATAAGGTCATGTA
core_57	TTGTTAAACGCTCTCCTGTTCCGACCCTGCCGGGGGTCGCAAATAGG
core_58	GCCGAAATCCAGGCGTTTCCCCCTGGAAGCTATTTACCATACATAT
core_59	AAGAATAGGGTGGCGAAACCCGACAGGACTAATATATGCATTTAT
core_60	TCCAGTTTGAGCATCACAAAAATCGACGCTCTTGATTATCATACT
core_61	AACGTGGGGCGTTTTTCCATAGGCTCCGCCCAATGTCAAGGAATAA
core_62	TTGCCCGGGCTGGCAAAAAGCAGCGCAAAACAGACTCG
core_63	CCACATAGGGAAGAAAATTCTTCATGCAATTGGAGCCT
core_64	TTGGAAAAGGAAAGCCCTTGTTGTAGTCGAGGATGCAT
core_65	AAGGATCTAAAGGGAAAAGCTTTGGTTGCTATCTCTAG
core_66	ATGTAACCGAGGTGCTGCTTTGCATACTTCTGCTGGGG
core_67	GGTGAGTACTGCAACTCCGGTGGATGGGAGGTGATGTCCAACTTGAT
core_68	ATGCCATTAATTGTGGCCGCTCTGTAGGCGCCGGGCAGCTGCACG
core_69	TATGGCATCGCCAGGCCCTCCTGGCCTTGTAGGTGGTCTTGACCT
core_70	GTAAGTTCCATTGCCAGTCGATAGTGGCCGCCGTCCTTCAGCTT

core_71	AGCTCCTTCGTCGTTTCAAACAAGCTTGATCTCGCCCTTCAGGGCG
core_72	GAGCGCAGCGGCTCCAAGTTGCTCGCACATTTTTAATATT
core_73	AAGTGGTCCTCAACCAGAACCTTTAGCGGCCGCATCCCCAGTTTAGT
core_74	CAGTCTATCCGTAAGAGCGAGCCAGGGGACTTTCCACACCGGATCTCT
core_75	TAAGTAGTGCACTGCATGCCTGCTGCTTTGCATACTTCTGCCTGCTGG
core_76	CGTTGTTGGGCCGCAGTGCTGTCCAGCTTTGGTTGCTGACTAATTGA
core_77	TCACGCTCGGTCCTAGAATGACAGCCTGGGGACTTTCCACACCGG
core_78	GCTTAATCTCACCTAGTCGCCCTTGCTATCCACGCCCATTGATGTAC
core_79	CCACCGCTCAGCAATAGCTGATGTCGGCGGGGTGCTTCA
core_80	CAAGCAGGAGACCCTTGGAGCCGAAGCCCTCGGGGAAG
core_81	TCTCAAGCCATCTGTGTCCCAGCGTCCTCGAAGTTCATGTTGACGT
core_82	GGTCTGACGTGTAGGGTCACCAGGAGTCCTGGGTCAGGCTTCTT
core_83	GTTAAGGGTTCGTTCATCGTAGGACCTTGTAGATGAACCAGCGTCG
core_84	AAGGATCTAGTGAGGCTCTCGAAGTCGGAGGGGAAGTTCAGCCTCT
core_85	AAATGAAGAACTTGGTCCATGTGCAGCCCATGGTCTTCTCCGTCCT
polyT_1	TTTTTAAAGGCCAGGAACCGTAAAAAGGCCGGCGAATGAAAAAACAG
polyT_2	TTTTTACGCTGTAGGTATCTCCGCTTTCTGCCTAACTCCTAAATT
polyT_3	TTTTTTTCGCTCCAAGCTGGGAGTTCGGTCAAACAAAACGGCTACACTAGTTTTT
polyT_4	CCGCATCACCATGGTAATTTTTTTTTTTAGCGATGACTAAGGCCAGCATTTTT
polyT_5	TTTTTGTTCAGCCCGACCAACAAAAAGAGTTGGTAGCTTTTT
polyT_6	TTTTTGGAGGCCTCCCACCTTGAAGCGTTTTTTTTTTCATGAACTCCT
polyT_7	TTTTTCTATCGTCTTGAGTCCAAGTTCCACGATGGTGTAGTGACAGCTT
polyT_8	AGACACGACTTTTTTTTTTTTTATCGCCACTGGCAGTGGACTTAGGG
polyT_9	GCGTTCGTAAGGGCCCAAGTAGTCGGGGCGCCTTATCCGGTAATTTTT
polyT_10	TTTTTTGGGAGTGGCACCGATCAAGGTTTTT
polyT_11	TTTTTCTTTGGTTGCTGACTCCGGATGGTTTAAAGTTTTT
polyT_12	TTTTTGAGCGAGGTATGTAGGACCACACCCGCAGTCAGCCACTGGTAACAGGATTAGCATTTTT
polyT_13	TTTCCACAAATTGAGAATTTTATTAGGAAAGGACAGTTTTT
polyT_14	TTTTTTCAGGGCGATGGCCCGCGAAAAACCGTCTATTTTT
polyT_15	TTTTTAAGGACAGTATTTACCCGGTGCTACAGAGTTCTTTTTTTTTTTTGAAGTGGTGCATAGCTCTTTTT
polyT_16	ATGATCCCTTCCCAACTTCCAGGCGGGGTACATCCGCTCGGATTTTT
polyT_17	TTTTTCGAGTTACTCACCAGCGTTTTTTTTTTTTTCTGGGTGAGC
polyT_18	TTTTTCAGTTACCTTCGGCAGTGCGCTCTGCTGAAGCTTTTT
polyT_19	TTTTTAAGTATATATGAGTATTTTAAATCAATCTATTTTT
polyT_20	TTTTTTCTTGATCCGGGTAGGTCGTTTTT
polyT_21	GGTAGCGGCCAGAGAGCTCTGTGCACGAACCCCCCTTTTT

3.4 sc_mCherry_6xSV40

Sequence sc_mCherry_6xSV40:
ggggatgcggccgctcgagtctagagATCCGGTGTGGAAAGTCCCCAGGCTCCCCAGCAGGCAGAAGTATG
CAAAGCATGCATCTCAATTAGTCAGCAACCAAAGCtctagagATCCGGTGTGGAAAGTCCCCAGGCT
CCCCAGCAGGCAGAAGTATGCAAAGCATGCATCTCAATTAGTCAGCAACCAAAGCtttaaaccATCC
GGTGTGGAAAGTCCCCAGGCTCCCCAGCAGGCAGAAGTATGCAAAGCATGCATCTCAATTAGTC
AGCAACCAAAGCtttaaacccgctgatcagcctcgactatacgcgttgacattgattattgactagttattaatagtaatcaattacggggtc
attagttcatagcccatatatggagttccgcgttacataacttacggtaaatggcccgcctggctgaccgcccaacgacccccgcccattgacgt
caataatgacgtatgttcccatagtaacgccaatagggactttccattgacgtcaatgggtggagtatttacggtaaactgcccacttggcagtac
atcaagtgtatcatatgccaagtacgccccctattgacgtcaatgacggtaaatggcccgcctggcattatgcccagtacatgaccttatgggac
tttcctacttggcagtacatctacgtattagtcatcgctattaccatggtgatgcggttttggcagtacatcaatgggcgtggatagcggtttgactca
cggggatttccaagtctccaccccattgacgtcaatgggagtttgttttggcaccaaaatcaacgggactttccaaaatgtcgtaacaactccgc
cccattgacgcaaatgggcggtaggcgtgtacggtgggaggtctatataagcagagctctctggctaactagagaacccactgcttactggctt
atcgaaattaatacgactcactatagggagacccaagctggctagcgtttaaacttaagcttggtaccgagctcggatccactagtccagtgtg
gtggaattcgccaccatggtgagcaagggcgaggaggataacatggccatcatcaaggagttcatgcgcttcaaggtgcacatggagggct
ccgtgaacggccacgagttcgagatcgagggcgagggcgagggccgcccctacgagggcacccagaccgccaagctgaaggtgacca
agggtggcccctgcccttcgcctgggacatcctgtcccctcagttcatgtacggctccaaggcctacgtgaagcaccccgccgacatccccg
actacttgaagctgtccttccccgagggcttcaagtgggagcgcgtgatgaacttcgaggacggcggcgtggtgaccgtgacccaggactcct
ccctgcaggacggcgagttcatctacaaggtgaagctgcgcggcaccaacttcccctccgacggccccgtaatgcagaagaagaccatgg

gctgggaggcctcctccgagcggatgtaccccgaggacggcgccctgaagggcgagatcaagcagaggctgaagctgaaggacggcgg
ccactacgacgctgaggtcaagaccacctacaaggccaagaagcccgtgcagctgcccggcgcctacaacgtcaacatcaagttggacat
cacctcccacaacgaggactacaccatcgtggaacagtacgaacgcgccgagggccgccactccaccggcggcatggacgagctgtac
aagtgagcggccgctcgagtctagagggcccgggtaaggagggcccgtttaaacccgctgatcagcctcgactgtgccttctagttgccagcc
atctgttgtttgcccctcccccgtgccttccttgaccctggaaggtgccactcccactgtcctttcctaataaaatgaggaaattgcatcgcattgtct
gagtaggtgtcattctattctggggggtggggtggggcaggacagcaagggggaggattgggaagacaatagcaggcatgctggggatgc
ggtgggctctatggctcgctttcttgctgtccaatttctattaaaggttcctttgttccctaagtccaactactaaactggggatgcggccgctcgagtc
tagagATCCGGTGTGGAAAGTCCCCAGGCTCCCCAGCAGGCAGAAGTATGCAAAGCATGCATCTC
AATTAGTCAGCAACCAAAGCtctagagATCCGGTGTGGAAAGTCCCCAGGCTCCCCAGCAGGCAGA
AGTATGCAAAGCATGCATCTCAATTAGTCAGCAACCAAAGCtttaaaccATCCGGTGTGGAAAGTCCC
CAGGCTCCCCAGCAGGCAGAAGTATGCAAAGCATGCATCTCAATTAGTCAGCAACCAAAGCtttaaa
cccgctgatcagcctcgactacaacaaggcaaggcttgaccgacaattgcatgaagaatctgcttagggttaggcgttttgcgctgcttgtgac
attaagcgcggcgggtgtggtggttacgcgcagcgtgaccgctacacttgccagcgccctagcgcccgctcctttcgctttcttcccttcctttctcg
ccacgttcgccggctttccccgtcaagctctaaatcgggggctccctttagggttccgatttagtgctttacggcacctcgaccccaaaaaacttg
attagggtgatggttcacgtagtgggccatcgccctgatagacggtttttcgccctttgacgttggagtccacgttctttaatagtggactcttgttcca
aactggaacaacactcaaccctatctcggtctattcttttgatttataagggattttgccgatttcggcctattggttaaaaaatgagctgatttaacaa
aaatttaacgcgaattttaacaaaatattaacgcttacaatttaggtggcacttttcggggaaatgtgcgcggaacccctatttgtttatttttctaaat
acattcaaatatgtatccgctcatgagacaataaccctgataaatgcttcaataatattgaaaaaggaagagtatgagtattcaacatttccgtgt
cgcccttattcccttttttgcggcattttgccttcctgtttttgctcacccagaaacgctggtgaaagtaaaagatgctgaagatcagttgggtgcacg
agtgggttacatcgaactggatctcaacagcggtaagatccttgagagttttcgccccgaagaacgttttccaatgatgagcacttttaaagttctg
ctatgtggcgcggtattatcccgtattgacgccgggcaagagcaactcggtcgccgcatacactattctcagaatgacttggttgagtactcacc
agtcacagaaaagcatcttacggatggcatgacagtaagagaattatgcagtgctgccataaccatgagtgataacactgcggccaacttact
tctgacaacgatcggaggaccgaaggagctaaccgcttttttgcacaacatgggggatcatgtaactcgccttgatcgttgggaaccggagct
gaatgaagccataccaaacgacgagcgtgacaccacgatgcctgtagcaatggcaacaacgttgcgcaaactattaactggcgaactactt
actctagcttcccggcaacaattaatagactggatggaggcggataaagttgcaggaccacttctgcgctcggcccttccggctggctggtttatt
gctgataaatctggagccggtgagcgtgggtctcgcggtatcattgcagcactggggccagatggtaagccctcccgtatcgtagttatctacac
gacggggagtcaggcaactatggatgaacgaaatagacagatcgctgagataggtgcctcactgattaagcattggtaactgtcagaccaa
gtttactcatatatactttagattgatttaaaacttcatttttaatttaaaaggatctaggtgaagatcctttttgataatctcatgaccaaaatcccttaac
gtgagttttcgttccactgagcgtcagaccccgtagaaaagatcaaaggatcttcttgagatcctttttttctgcgcgtaatctgctgcttgcaaaca
aaaaaaccaccgctaccagcggtggtttgtttgccggatcaagagctaccaactctttttccgaaggtaactggcttcagcagagcgcagatac
caaatactgtccttctagtgtagccgtagttaggccaccacttcaagaactctgtagcaccgcctacatacctcgctctgctaatcctgttaccagt
ggctgctgccagtggcgataagtcgtgtcttaccgggttggactcaagacgatagttaccggataaggcgcagcggtcgggctgaacggggg
gttcgtgcacacagcccagcttggagcgaacgacctacaccgaactgagatacctacagcgtgagctatgagaaagcgccacgcttcccga
agggagaaaggcggacaggtatccggtaagcggcagggtcggaacaggagagcgcacgagggagcttccagggggaaacgcctggt
atctttatagtcctgtcgggtttcgccacctctgacttgagcgtcgatttttgtgatgctcgtcaggggggcggagcctatggaaaaacgccagca
acgcggcctttttacggttcctggccttttgctggccttttgctcactcatt

Corresponding 20HB DNA origami design and staple list for sc_mCherry_6×SV40:

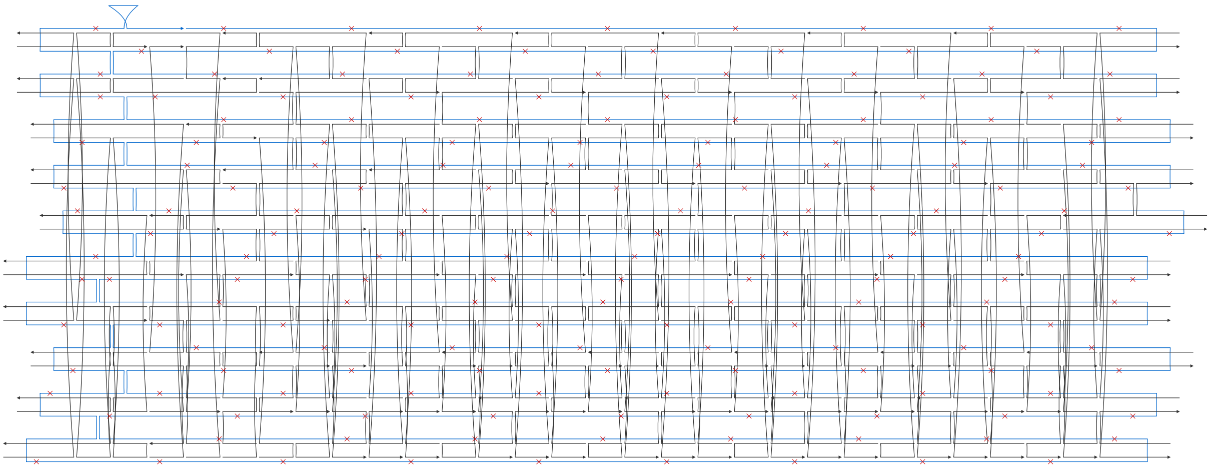

Fig. S12 | Scaffold routing and staple design for 20HB_6×SV40. Scaffold routing is shown in blue, staples are given in black, skips are given as red crosses. Design was prepared using caDNAno v0.1.

Table S7 | Individual staple sequences for sc_mCherry_6×SV40 20HB.

Name	Sequence 5' - 3'
core_1	CGTAATTGTGGAAAGTCCCTATTTTTGGT
core_2	AATCAATGTTACCGTAAATACTCTCAATGGG
core_3	TCAGCGGGGATACACTTGATGTAAGTCAAAC
core_4	ATTGAGATTGACGTCAATAGGGCTGCCAAA
core_5	GCTGGGGGGCATAATGCCAGGCGATGACTAA
core_6	GGTCGTTGGGCGGTCAGACTACCGCCCATTTGCGTCAATGGGGCGGAACGTCAAT
core_7	TATGGGATATGGGCTATGAACTGGGGACTT
core_8	GACGTCAAATTACTATTAATAACGCTTTGCA
core_9	TGGGCAGTTCAACGCGTATAGTCGAGCTTTG
core_10	TGGCATATTTTAAAGCTTTGGTTGAGCCTGG
core_11	TACCGTCATGCATGCTTTGCATACATGCATGC
core_12	GTTGTTAGAGCTCTGCTTATATGGGTGCTT
core_13	CAGTAAGCAGTGGGTTTGAACTGAATGAGATTCACCTATC
core_14	CCTATAGTGAGTCGTAGGGCAGGGGAACGAAAATCCATAG
core_15	TTAAGTTTAAACGCTATTGGCGGTTGATCTTTATAACTAC
core_16	GGACTAGTGGATCCGCCTCGCCCGCAGAAAGCCCCAGT
core_17	TGCTCACCATGGTGGGCCGTTCGGTTTTTACGCTCAC
core_18	ATCACGCCCTTGGAGCCGTACACTCTAGTGACTTATC
core_19	CACGGTCGATGTCCCAGGCGAATTAATTTACAGGATT
core_20	ACTCGCCGCTTGGTCACCTTCAGCGCCAGCTTTGCTACAG
core_21	TTGGTGCCCCCTCGTAGGGGCGGCAGCTCGGTTACGGCT
core_22	CTTCTGCACGATCTCGAACTCGTGCGAATTCCTCTGCGC
core_23	GCTCGGAGCTCCATGTGCACCTTTTATCCTCGAAAAAGA
core_24	GTGGAGTTAGACTCGAGCGGCCGATACTTCT
core_25	TGTTCCACGGTTTAAACGGGCCCTTTCCACAC
core_26	CGGCCCTCGGCGGCCCTCGGCGCGTTCGTACCGAAGTTCAGTGAGGATCAAAAGAAGTAA
core_27	ATCAGCGGATGGTGTAGTCCTCGTTGTGGGTCCTGGGTTTCGTTCACTCACGTGGTTATG
core_28	AACTAGAAGGCACAGTGCATCCCCTGGTGTCA
core_29	GGCTGGCTCCAACTTGATGTTGACGTTGTAGTAGATGATCGTGTAGTCTACGGGTGTCATGC
core_30	CGGGGGAGGGGCAAAACAAAGGCGCAACGT
core_31	GGAAGGCAGCAGCTGCACGGGCTTCTTGGCCAGGGGAAGACCATCTGAAAAGGATACTGG TGA
core_32	GGAGTGGCACCTTCCAAGAAAGAGAGTAA
core_33	GGACAGTGGGTCTTGACCTCAGCGTCGTAGTATGGTCTTCGAGACCCTTGTTTGCAATAGTG T
core_34	AATTTCCTCATTTTACCCAGCATCATCCAG
core_35	TGCGATGCTCCTTCAGCTTCAGCCTCTGCTTTACATCCCAGCAATAAAACAAACCCCGGCGT
core_36	TCCCCCTTGCAGAAGTTGTTGTAGCCCTTATAAATCAAAACTCATTTAAAGTGCT
core_37	TCTCTAGAGGGAGCCTGGGGACTCCTTACCGCTTAATC
core_38	GCTGGGGACTAGACTCGAGCGGCCCGAGGCTGCTGTCTAT
core_39	ATTGAGATTAGTTGGACTTAGGGACAACAGATCTCCCCG
core_40	CCGGATGGATAGAAATTGGACAGCAGGGTCAAAGGGCTT
core_41	TGCCTGCTAGAGCCCACCGCATCTTAGGAAATGATACCG
core_42	ACTAATTTTGTCTTCCCAATCCTCAGACAAAGATTTAT
core_43	CTGGCAAGGCCCACTACGTGAAATATTTGACCAGCGT
core_44	ACCACCACCAAAGGGCGAAAAACTAGGGGTTCCCAACTG
core_45	AGCAGCGCGTCCACTATTAAAGAAGCCACCTAGAGATCCA
core_46	TCTTCATGGGGTTGAGTGTTGTTCTAAAATTCGGCGAAAA
core_47	AGGGAGCCAGTGTGCAAAAAAGCGAATGAAGT
core_48	ATATTATTGAAGCATGGTGCCGTCCTCCGATCAACGATTTACCAAT
core_49	CTCATGAGCGGATACCCATCACCCAGTGTTAGTTTGGTTCAGCGAT
core_50	AGAAAAATAAACAAACGTCTATCGCATAATTTTGCTACATTGCCTGA
core_51	ATTTCCCCGAAAAGTCGTGGACAGATGCTTCCAGTTAAGATACGGG
core_52	AGCGTTAATATTTTGTCAGTTTGCCAAGTCTTGTTGCCGCTGCAA
core_53	ATTTTTGTTAAATCAGGAATAGACACCGAGTCAACTTTACGGCTCC
core_54	AATGTTGACGCCTTATTTTAAATCAGAAGTAGTCGGGGATGTCGGCGAGACCTCC
core_55	GCAAAATACTCCATAACATACGTCATTATTG
core_56	ACAGGAAGGTTATTGTTCCACACCGGATCTCTAGACTCGACGCGGAGCCGCAAA

core_57	GAGCAAAAGTTGGCCGCTAATCAAGCGAAAGGAGCGGGCG
core_58	TACTTTCAATGTATTTTACTTCTGCCTGCTGGGGAGCCTAATGACCCTTCTGGGTCAGCAGC
core_59	GCATCTTTGCAGCACTAGGGCGATGTGTAGCGGTCACGCT
core_60	CTCGTGCACCGCGCACGTTGCTGACTAATTGAGATGCATTAGTCAATATCTTCACGAGGTAT
core_61	GTAACCCACATCCGTATCCAACGTACCCGCCGCGCTTAAT
core_62	CCGCTGTTAATTGTAGGACTTTCCACACCGGATCTCTAGAGGCTGAGTTCGATGAAGTGGT
core_63	GATCTTAGTACTCAAGAACAAGAAAAACGCCTAACCCT
core_64	TTCTTCGGGCGTTAATTTGCATACTTCTGCCTGCTGGGGCTGACTACTCTCAAGAGGACAGT
core_65	GAAAACGATGCGGCGCGAGATACAATTGTCGGTCAAG
core_66	AACTTTATTTAACCAGCTTTGGTTGCTGACTAATTGAGTTCTGCCTCATCATTGAGCCAGTT
core_67	GTTACATGGAAAGCCGAAATCGGAACCCTAAACTCTTCGACACGGAGGGCCATT
core_68	ATTCAGCTGAAGAAAGTTTTTTGGGGTCGATTATCAGG
core_69	ATGGCTTCCGGATCTGCCTGGGGACTTTCCACACCGGAAAGGAAGGCCGGTTCCCGTTGTCA
core_70	GGCATCGAGTTTAGGCATGCTTTGCATACTTCTGCCTCTAGGGCGCGCTCGTCTCACTCAT
core_71	TAGTTTGAACCTTTATTTAAAGCTTTGGTTGCTGACTAGCGCGTATGTTGCCACTCTTAC
core_72	GGGAAGCTCGAGCCATGGGGAGCCTGGGGACTTTCCACAGTCACAAGTAGTTCGTTCTGTG
core_73	TCCGCCTCGCCTGCTAGAGATGCATGCTTTGCATACTTCAAGCAGATTCTATTAAATTCTGAG
core_74	AATTAAAGTTAGCTCTAAGGGCCTTTTTCAGCATCCCCAATGAGTGAGCAAGCAGCCAGGC
core_75	TCCTTTTACTGACAGCAAGGCGAGCCTGCTGGCTTTGGTTGCTGACTAATTGAGTTGACGGG
core_76	AGGATCTTTAAGACACTAGCCAGACGACATTTTGGAAAGTCCCGTTGAGGCGTTACGCCACTGG
core_77	TTTTGGTCGGGGACAGACCACGCCGCCGTCCT
core_78	TAAGGGACACTGGTACGATAAGCGCCAAAACAAACTCCCATTGACGCACCCATTAGCAGAG
core_79	GCTCAGTGGGCCACCTCCTGCAGGGAGGAGAGGTGATG
core_80	GTCTGACGTAGGCGGGGGTCTCGTGGAGACTTGGAAATCCCCGTGCTGCCAAGAGTTCTT
core_81	GATCCTTCTGGGTGGCGCAGCTTCACCTTGGCGCCGG
core_82	CTCAAGAAGGCCTAACACCAAGCCGCTATCCACGCCCATTGATGTAGGCGTACTACACTAGA
core_83	GATTACGCTCGCCCTTTACGGGGCCGTCGGTTGTAGGT
core_84	AAGCAGCAATTTGGTAACCACACTACCGCATCACCATGGTAATAGCGGGCCATTTCTGCTGA
core_85	GTAGCGGTACGGAGCCGAGGCCTCCCAGCCCGGCCGCCG
core_86	CTATCGTCGGGCGGGTACCGTAAGTTATGTAAGCGGCC
core_87	TTGAGTCCAAAGGGAACTTCGGTAAAGCACTGCGAACGTGGCGAGA
core_88	AACCCGGCACCTAGACACGTAGGGCTCCCACTTGAAGCC
polyT_1	TTTTTGGATGGTTTAAAATAGGCCGAAATTTTT
polyT_2	TTTTTTAAAAAGGCCGCGTTGAAAGGCCGCGCTTTCATACTCAT
polyT_3	ATGTACTGAGCCTGGGGACTTTCCACACCTTTTT
polyT_4	TTTTTTCCCATAAGGTCGTTGGTAGCTCTTTTT
polyT_5	TTTTTAGGCTCCGCCCCCCTCTGGCGTGACCGCTGTCATAGCTCACGCTTTTT
polyT_6	CTCGCCCTTACGTAGATGTACTGCCAAGTAGGAAAGTTTTT
polyT_7	TTTTTTTGATGATGGCCATGGAAGCGCATGAACTCCTTTTT
polyT_8	TTTTTGACGCTCAAGTCAATGTGTGCACGAACCCCCTTTTT
polyT_9	TTTTTGGCGCCGTCCTCGGGGGATCTCGTAGAATGACACCTAC
polyT_10	TTTTTAGGACTATAAAGATACCCAGCTCGTCCATGCCGCCGCTCGGGGAACTTGGT
polyT_11	TTTTTACCCCCCAGAACCCTTCAGTTTTT
polyT_12	TTTTTGAAGCTCCCTCGTGCGCATGCTTTGCCTCACTT
polyT_13	TTTTTTCAGCGGGTTTAAAGCTTTGGTTGCTGCCTTGCCTGGTCCTGTGCTCTTGCACCGCTGACCTTCG
polyT_14	TTTTTTTACCGGATACCTGTGATTTAGAGCATGCTCTCCTGTTCCGACCCTGCCGCTTTTT
polyT_15	TTTTTTCGGCAAAATTCGAGGCTGATTTTT
polyT_16	TTTTTGGGAAGCGTGAGGAACCGTTTTT
polyT_17	TTTTTCCGCGCCACATAGCAGCAATACGGGATAATATTTTT
polyT_18	TTTTTTGTAGGTATCTCCCCCCGCCTTTCTCCCTTCTTTTT
polyT_19	ATCCCCCATGTTTCGAAAGTATAGTAAGGCGTTTCCCCCTGTTTTT
polyT_20	TTTTTGGAAGGGCCGAGCGCTGTCCTGCCCCACCCCTTTTT
polyT_21	TTTTTTCCAAGCTGGGCTCTGTGTAGGTCGTTCGCTTTTT
polyT_22	TATGAGTAAAGGACAGCTTCAGGTGGCGAAACCCGACTTTTT
polyT_23	TTTTTTTGATCCGGCAACCAGCCAGCCTTTTT
polyT_24	TTTTTCGTTCAGCCCTTTTCCATTTTTT
polyT_25	CCGGTAACACCGTACACGCCGAGCATCACAAAAATCTTTTT
